# YME1L-mediated mitophagy protects renal tubular cells against cellular senescence under diabetic conditions

**DOI:** 10.1186/s40659-024-00487-0

**Published:** 2024-03-17

**Authors:** Yuanyuan Luo, Lingxiao Zhang, Ning Su, Lerong Liu, Tongfeng Zhao

**Affiliations:** 1 Department of Endocrinology, The Sixth Affiliated Hospital, Sun Yat-Sen University, Guangzhou, 510655 China; 2 Department of Endocrinology, Chongqing University Three Gorges Hospital, Chongqing, 404000 China; 3 Department of Hematology, The Sixth Affiliated Hospital, Sun Yat-Sen University, Guangzhou, 510655 China; 4 Department of Endocrinology, Southern Medical University Nanfang Hospital, Guangzhou, 510515 China; 5 Department of Endocrinology, The Third Affiliated Hospital of Southern Medical University, Guangzhou, 51000 China

**Keywords:** Diabetic kidney disease, Tubular cell, YME1L, Cellular senescence, Mitophagy

## Abstract

**Background:**

The senescence of renal tubular epithelial cells (RTECs) is crucial in the progression of diabetic kidney disease (DKD). Accumulating evidence suggests a close association between insufficient mitophagy and RTEC senescence. Yeast mitochondrial escape 1-like 1 (YME1L), an inner mitochondrial membrane metalloprotease, maintains mitochondrial integrity. Its functions in DKD remain unclear. Here, we investigated whether YME1L can prevent the progression of DKD by regulating mitophagy and cellular senescence.

**Methods:**

We analyzed YME1L expression in renal tubules of DKD patients and mice, explored transcriptomic changes associated with YME1L overexpression in RTECs, and assessed its impact on RTEC senescence and renal dysfunction using an HFD/STZ-induced DKD mouse model. Tubule-specific overexpression of YME1L was achieved through the use of recombinant adeno-associated virus 2/9 (rAAV 2/9). We conducted both in vivo and in vitro experiments to evaluate the effects of YME1L overexpression on mitophagy and mitochondrial function. Furthermore, we performed LC–MS/MS analysis to identify potential protein interactions involving YME1L and elucidate the underlying mechanisms.

**Results:**

Our findings revealed a significant decrease in YME1L expression in the renal tubules of DKD patients and mice. However, tubule-specific overexpression of YME1L significantly alleviated RTEC senescence and renal dysfunction in the HFD/STZ-induced DKD mouse model. Moreover, YME1L overexpression exhibited positive effects on enhancing mitophagy and improving mitochondrial function both in vivo and in vitro. Mechanistically, our LC–MS/MS analysis uncovered a crucial mitophagy receptor, BCL2-like 13 (BCL2L13), as an interacting partner of YME1L. Furthermore, YME1L was found to promote the phosphorylation of BCL2L13, highlighting its role in regulating mitophagy.

**Conclusions:**

This study provides compelling evidence that YME1L plays a critical role in protecting RTECs from cellular senescence and impeding the progression of DKD. Overexpression of YME1L demonstrated significant therapeutic potential by ameliorating both RTEC senescence and renal dysfunction in the DKD mice. Moreover, our findings indicate that YME1L enhances mitophagy and improves mitochondrial function, potentially through its interaction with BCL2L13 and subsequent phosphorylation. These novel insights into the protective mechanisms of YME1L offer a promising strategy for developing therapies targeting DKD.

**Supplementary Information:**

The online version contains supplementary material available at 10.1186/s40659-024-00487-0.

## Introduction

Diabetic kidney disease (DKD) stands as one of the most severe complications of diabetes and has emerged as the primary cause of end-stage renal disease (ESRD) globally [1]. Recent research has found that the pathogenesis of DKD involves several factors, including the downregulation of endothelial cell glucocorticoid receptor leading to epithelial-mesenchymal transition (EMT) [2], abnormal activation of the Wnt signaling pathway [3], the TGF-β signaling pathway [4], and dipeptidyl peptidase-4 (DPP-4)-mediated signaling mechanism [5, 6], while some drugs such as angiotensin-converting enzyme inhibitors (ACEi), angiotensin receptor blocker (ARB), and sodium-glucose co-transporter-2 (SGLT2) inhibitors, as well as mineralocorticoid receptor antagonist finerenone [7], have been used in efforts to slow the progression of DKD or improve renal function, but existing therapies have not effectively halted the advancement of DKD. This lack of progress leaves 20%-40% of individuals in need of renal transplantation [8]. Therefore, the search for novel therapeutic and preventive strategies for DKD is of utmost importance.

Extensive research has consistently shown that renal tubulointerstitial fibrosis (TIF) plays a pivotal role in the advancement of DKD, the severity of TIF is closely linked to renal impairment, which in turn serves as a prognostic indicator for DKD [9]. Among its constituents, the renal tubular epithelial cell (RTEC) stands out as a primary component, and its functional impairment is intricately associated with TIF [10, 11]. Upon persistent exposure to high-glucose (HG) ambiance, RTECs may sustain dysfunction or injury following activation of the signaling of cellular senescence, which in turn leads to tubular injury via a pro-inflammatory response known as senescence-associated secretory phenotype (SASP) [12]. SASP encompasses the release of diverse molecules, such as cytokines, chemokines, and growth factors. It has been proposed as a significant contributing factor to promote TIF [13, 14]. Therefore, it is necessary to clarify the role and mechanism of RTEC senescence in the pathogenesis of DKD.

Mitophagy, the selective degradation of mitochondria by autophagy, maintains cell homeostasis and ensures mitochondrial homeostasis via specifically degrading damaged mitochondria [15]. Studies have shown that the downregulation of mitophagy in RTECs is closely related to RTEC senescence in response to DKD [16]. Insufficient mitophagy leading to the accumulation of mitochondrial fragments results in the excessive generation of mitochondrial reactive oxygen species (mtROS), which play critical roles in cellular senescence by inducing genomic damage [17]. Identifying reliable effector molecules of mitophagy may provide a target for therapies against RTEC senescence and tubular injury in DKD.

The ATPases Associated with diverse cellular Activities (AAA) family is a group of ATPases belonging to the cellular protease system [18]. Recent studies have shown that targeting AAA family proteins can significantly delay the occurrence and development of the aging process and hinder age-related chronic diseases [19–21]. Yeast mitochondrial escape 1-like 1 (YME1L), a member of the AAA family, is a nuclear genome-encoded metalloprotease embedded in the inner mitochondrial membrane [22], YME1L also has chaperone-like activity involved in the recognition and interaction with other proteins in addition to its protease activity [23]. YME1L has recently been reported to have multiple effects on mitochondrial protection in response to several types of stress, such as oxidative stress [24], heart failure [25], and hypoxia [26]. Nevertheless, very little is known about the potential role of YME1L in regulating mitochondrial function, mitophagy, and cellular senescence in DKD.

In the present study, we found that YME1L expression was downregulated in the renal tubules in DKD patients and high-fat diet (HFD)/Streptozotocin (STZ)-induced DKD mice, restoration of YME1L expression in RTECs significantly alleviates RTEC senescence and improves renal function. Mechanistically, YME1L reconstructs the mitophagy of RTEC by interacting with the mitophagy receptor BCL2 like 13 (BCL2L13). Hence, YME1L may be considered a therapeutic target to prevent DKD progression.

## Materials and methods

### Patients

Human kidney biopsy samples from patients with DKD and Lupus nephritis (LN) were obtained from the Department of Pathology, The Sixth Affiliated Hospital of Sun Yat-sen University. Normal kidney tissues from nephrectomies of renal hamartomas were collected as controls. Human subject data are summarized in Additional file 1: Table S1. The protocol of the present study was approved by the ethical committee of the Sixth Affiliated Hospital of Sun Yat-sen University.

### Animals

**Fig. 1:**
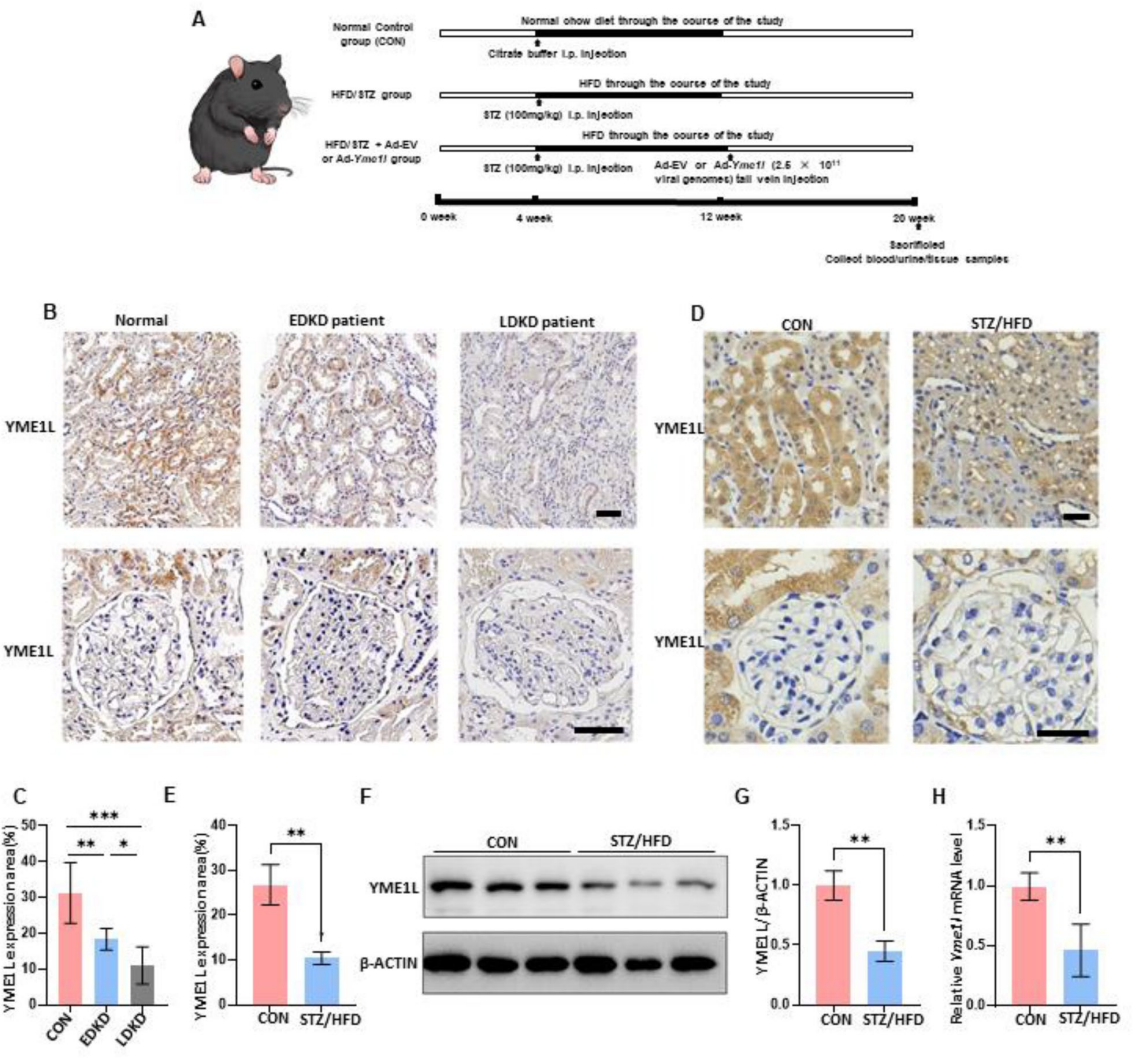
Expression pattern of YME1L in kidneys from patients and mice with DKD. **A** Experiment schedule of the mouse model constructed in this study. **B** Representative immunohistochemical staining of YME1L in renal tissues from normal subjects (Normal), EDKD and LDKD patients and (**C**) the percentage of YME1L expression area was quantified (n = 6–11). Scale bar: 40 µm. **D** Representative immunohistochemical staining of YME1L in renal tissues from CON and HFD/STZ mice, and **E** the percentage of YME1L expression area was quantified (n = 4). Scale bar: 20 µm. **F**, **G** Western blotting and quantitative analysis of kidney YME1L expression in CON and HFD/STZ mice (n = 6). **H** RT-PCR analysis of kidney *Yme1l* mRNA expression in CON and HFD/STZ mice (n = 4). Data are shown as mean ± SD. **p < 0.01

4-week-old male C57BL/6 mice were purchased from the Charles River Company (Beijing, China). Mice were randomly divided into 4 groups as follows: Group 1: Normal mice received citrate buffer on the day of injection STZ (CON); Group 2: Diabetes group (HFD/STZ), mice received a high-fat diet and injection of STZ; Group 3: Mice received a tail-vein injection of recombinant adeno-associated virus 2/9 (rAAV 2/9) control vector after STZ injection (HFD/STZ + Ad-EV); Group 4: Mice received a tail-vein injection of rAAV 2/9 vector expressing YME1L after STZ injection (HFD/STZ + Ad-*Yme1l*). The protocol of present study was approved by the ethical committee of the Sixth Affiliated Hospital of Sun Yat-sen University. The experiment schedule of the mouse model is shown in Fig. 1A.

### DKD mice

HFD/STZ were used to produce the DKD mouse model [27]. The control group were fed a normal chow diet, and the experimental groups were fed an HFD. After 4 weeks, experimental model mice were i.p. injected with a dose of STZ (100 mg/kg) to generate diabetes. 1 week later, the mice were considered diabetic when their random blood glucose levels exceeded 16.7 mmol/L. The control group received citrate buffer and were processed in parallel with the diabetic mice.

### Adeno-associated-virus-treated mice

rAAV 2/9 carrying cytomegalovirus (CMV)-enhancer-*Ksp-cadherin*-promoter-*Yme1l*-HA (Ad-*Yme1l*) was used to construct a tubule-specific *Yme1l* overexpression mouse model as previously described by Genomeditech (Shanghai, China) [28]. In brief, 1342 bp of the 5’ flanking regions of *Ksp-cadherin*, which is a unique, tissue-specific member of the cadherin family that is exclusively expressed in RTECs [29], was used as the upstream promoter of *Yme1l*, then combined this promoter sequence with the CMV-enhancer to enhance transcriptional activity, HA tag was fusion as a reporter gene at the C-terminus. The control vector was prepared as a negative control sequence (Ad-EV). The mice received a single injection of Ad-EV, and Ad-*Yme1l* at a dose of 2.5 × 10^11^ viral genomes through the tail vein.

### Cell culture and treatments

The human proximal tubular cell line HK2 was kindly provided by Stem Cell Bank, Chinese Academy of Sciences (Shanghai, China) and cultured in normal glucose medium (NG) (5.5 mmol/l glucose) supplemented with 10% fetal bovine serum (ExCell Bio, Jiangsu, China). For HG treatment, HK2 cells were exposed to a culture medium supplemented with 30 mM D-glucose at indicated time points. To inhibit mitophagy, HK2 cells were pretreated with 5 μM Mdivi-1 for 6 h before exposure to D-glucose. For gene disruption, HK2 cells were transfected with siRNA and plasmids using Lipofectamine 3000 (Invitrogen, Waltham, MA, USA) following the manufacturer’s protocol. The siRNA target sequences are detailed in Additional file 1: Table S2.

### Histology

Paraffin-embedded kidney sections were subjected to staining with H&E, SIRIUS RED, and MASSON using commercially available kits (Servicebio, Wuhan, China) for histological analysis.

### Immunohistochemistry

Paraffin-embedded kidney sections were deparaffinized and rehydrated with xylene and alcohol, followed by antigen retrieval under high-pressure buffer. Then, slides were incubated in 3% hydrogen peroxide to block endogenous peroxidase activity. They were then blocked by 5% normal goat serum, followed by incubation with primary antibodies at 4 °C overnight. Slides were further incubated with the HRP-conjugated second antibody, the immunoreactivity was detected by diaminobenizidine and the nuclei were counterstained with hematoxylin. Images were captured by a slide scanning imaging system (TEKSQRAY, Shenzhen, China).

### Immunofluorescence staining

For immunofluorescence, cells were fixed with 4% paraformaldehyde, then permeabilized and blocked with 0.1% Triton X-100 (Sigma-Aldrich, St Louis, MO, USA) and 5% BSA (Biotopped, Beijing, China). Cells were incubated with primary antibodies at 4 °C overnight, followed by the fluorescent-labeled secondary antibody. The nuclei were stained with DAPI (Sigma-Aldrich, St Louis, MO, USA). Labeled cells were visualized and imaged under a confocal microscope (LSM880, ZEISS, Germany).

### Transmission electron microscopy (TEM)

The autophagic structures were observed using a TEM (Hitachi HT7700, TO, Japan). In brief, several 1 mm cubes from the renal cortex were cut and fixed with 4% glutaraldehyde. The samples were dehydrated through a graded ethanol series, then incubated in 100% ethanol and propylene oxide as well as two exchanges of pure propyleneoxide. Then they were embedded in epoxyresin and polymerized at 60 ˚C for 48 h. Specimens were cut into 70–80 nm ultra-thin sections, then mounted on 300-mesh copper grids. Sections were stained with uranyl acetate and leas citrate, then observed.

### Western blotting

Kidney tissues or HK2 cells were collected and homogenized with RIPA buffer. Protein was separated by gel electrophoresis, transferred to a poly-vinylidene fluoride membrane, and blocked with 5% milk. The membrane was incubated overnight with primary antibodies at 4 °C (Additional file 1: Table S3). Then, the membranes were incubated with an HRP-conjugated second antibody. A ChemiDoc Imaging System (Bio-Rad, CA, USA) was used for analyzing immunoreactive bands. The results were quantified by ImageJ (Rawak Software, Stuttgart, Germany).

### RT-PCR

Total RNA was extracted from the samples using RNAiso Plus (Takara, Otsu, Japan). cDNA was generated using a NovoScript Plus All-In-One 1st Strand cDNA Synthesis Kit (Novoprotein, Shanghai, China). RT-PCR was implemented using NovoStart SYBR qPCR SuperMix Plus (Novoprotein, Shanghai, China). The primer sequences are shown in Additional file 1: Table S4.

### RNA sequencing

RNA sequencing was performed by Geneseed (Guangzhou, China) following standard protocols. Qualified libraries were sequenced on Novaseq 6000 PE150 pattern. Bioinformatics analysis was conducted by R version 4.0.2 (https://www.r-project.org/). The differentially expressed genes (DEGs) between the normal and DKD was implemented via the “limma” R package [30] and visualized with heatmaps and volcano plots by the “ggplot2” package in R software [31]. Gene set enrichment analysis (GSEA) [32] was also performed to identify the underlying pathways, and the threshold for significant terms was adjusted* p*—value < 0.05.

### Flow cytometry analysis

For ROS detection, HK2 cells were incubated with DCFH-DA. For cell cycle detection, HK2 cells were incubated with DNA staining solution together with permeabilization solution (MultiSciences, Hangzhou, China). The fluorescent signal was detected by flow cytometry (CytoFLEXS, Beckman, Brea, USA).

### MitoSOX assay

HK2 cells were incubated with MitoSOX Red Mitochondrial Superoxide Indicator (YEASEN, Shanghai, China), then visualized and imaged under a confocal microscope (LSM880, ZEISS, Germany).

### The quantification of senescence-associated β-galactosidase activity (SA-β-Gal)

Cells or frozen sections were fixed, and the SA-β-Gal staining assay was carried out using SA-β-Gal Staining Kit (Beyotime, Shanghai, China).

### Co-immunoprecipitation (Co-IP)

Co-IP was performed using Protein A/G Magnetic Beads (Biolinkedin, Shanghai, China) according to the manufacturer’s protocol. In brief, HK2 cells lysed into cell lysates and incubated with the corresponding antibody for immunoprecipitation (IP) at 4 °C overnight. 50 µL of protein A/G beads were transferred to the protein-antibody complexes, and immunoprecipitates were collected after 2 h incubation. The pellet was suspended in 1 × SDS sample buffer, boiled, and subjected to SDS-PAGE for western blotting analysis.

### LC–MS/MS analysis for YME1L-interacting proteins

LC–MS/MS analysis was performed at Fitgene Biotechnology Co. (Guangzhou, China). In brief, after separating in SDS-PAGE gel and performing silver staining, gels were collected and digested. Residing peptides were extracted and dissolved in 2% acetonitrile and 0.1% formic acid. A liquid chromatography assay was performed using Acclaim PepMap RSLCC18m and Acclaim PepMap 75 µm × 150 mm (Thermo Fisher Scientific, Waltham, MA, USA). Separated peptides were analyzed with the mass spectrometer (Thermo Fisher Scientific, Waltham, MA, USA). Bioinformatics analysis was conducted by R software.

### ATP assay

ATP levels were detected using the Enhanced ATP Assay kit (Beyotime, Shanghai, China).

### ELISA

Cell supernatant IL-6 and TGF-β1 levels were determined by human ELISA kits (NeoBioscienceTechnology, Shenzhen, China).

### Statistical analysis

All the quantified data are expressed as the means ± SD. An unpaired Student’s t-test was performed to analyze the differences between the two groups. For multiple groups, one-way ANOVA was carried out, followed by the Bonferroni post hoc test. Statistical analyses were performed with GraphPad Prism 9.0 (GraphPad Software, La Jolla, CA, USA). A value of *p* < 0.05 was considered significant.

## Results

### YME1L expression is decreased in kidneys of DKD

To preliminarily assess the potential involvement of YME1L in DKD, we analyzed its expression in the kidneys of DKD patients. This includes patients in pathological stage II of early-stage DKD (EDKD), as well as those in the late stages of DKD (LDKD), specifically stages III-IV of advanced DKD. Immunohistochemical staining revealed that YME1L levels were lower in the renal tubules of DKD patients compared to those of normal subjects, additionally, there was a slight decrease in the early stages, and a significant decrease in the later stages, while YME1L expression in the glomerular area was less affected in the diabetic state(Fig. 1B, C). Furthermore, the expression of YME1L in patients with Lupus Nephritis (LN) showed no significant changes, indicating a unique role of YME1L in DKD (Additional file 1: Figure S1A, B). Similarly, YME1L expression was reduced in the renal tubules of HFD/STZ-induced diabetic mice compared to control mice (Fig. 1D, E). Western blotting and RT-PCR were used to further confirm the change in YME1L expression. The protein and mRNA levels of YME1L were significantly decreased in the kidneys of HFD/STZ-induced diabetic mice compared to control mice (Fig. 1F-H). These findings suggest that YME1L may play a role in renal tubular function and the progression of DKD.

### YME1L overexpression prevents renal dysfunction in diabetic mice

To test whether YME1L directly influenced the progression of DKD, diabetic mice were injected with Ad-*Yme1l* or Ad-EV, the rAAV-mediated strong transgene expression of HA tag was observed only in tubules, but not in the glomeruli (Additional file 1: Figure S2A). Furthermore, the overexpression of YME1L in renal tubules validated via western blotting, RT-PCR, and immunohistochemical staining (Additional file 1: Figure S2B-F). The urinary albumin-to-creatinine ratio (UACR) levels significantly decreased after YME1L overexpression (Additional file 1: Figure S3A). However, there was no significant difference in blood glucose, serum creatinine, serum urea, and lipid parameters among diabetic mice transfected with Ad-*Yme1l* and Ad-EV (Additional file 1: Figure S3B-G).

**Fig. 2:**
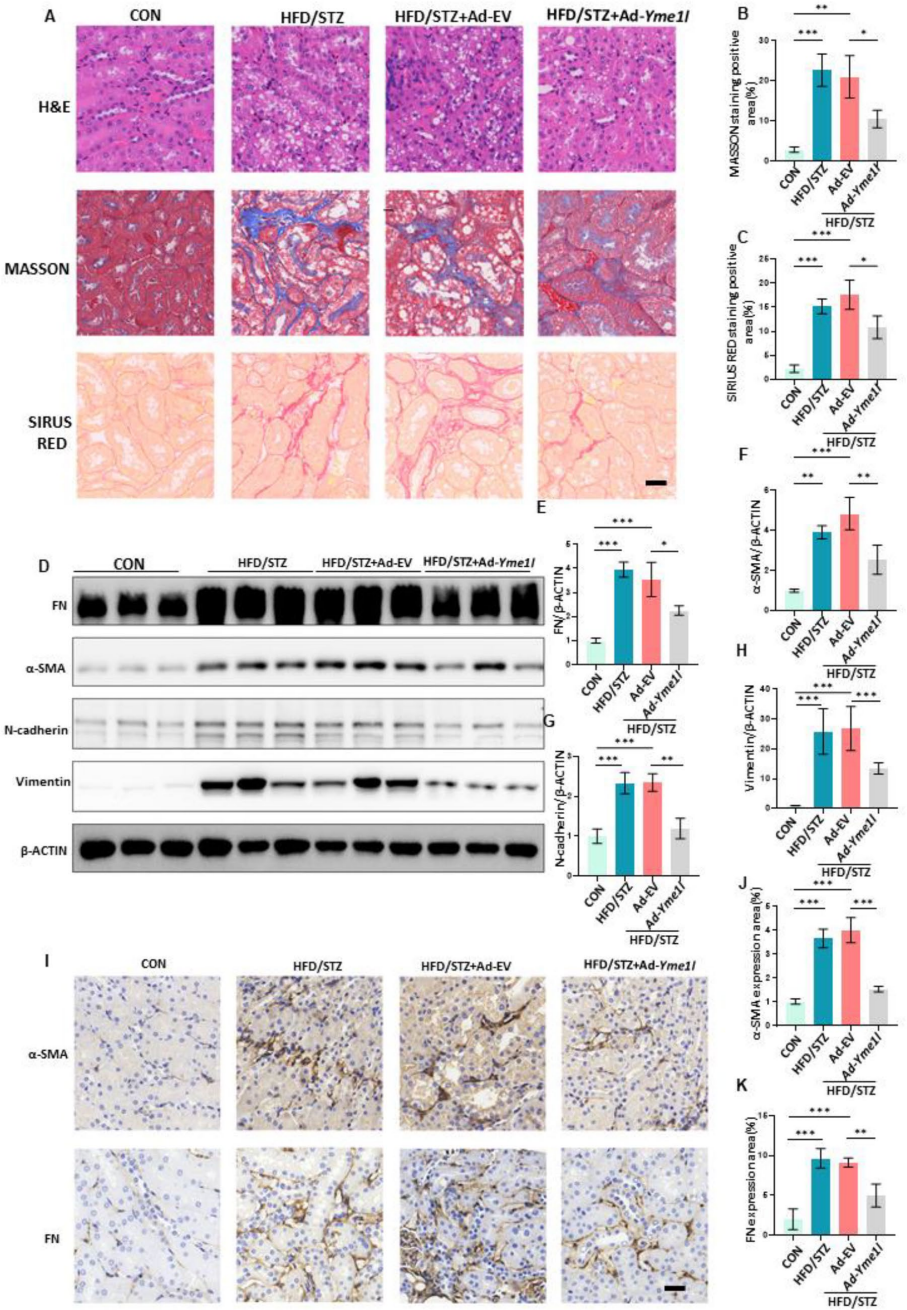
YME1L overexpression prevents renal tubule injury in diabetic mice. **A** Representative images of H&E staining, MASSON staining, and SIRIUS RED staining and **B**, **C** the percentage of MASSON staining positive area and SIRIUS RED staining positive area were quantified (n = 4). Scale bar: 20 µm. **D**-**H** Western blotting and associated quantitative analysis of kidney α-SMA, FN, N-cadherin and vimentin expression in each group (n = 4). **I** Representative immunohistochemical staining showed the tubular expression of α-SMA, FN, and **J**, **K** the percentage of α-SMA, FN expression area was quantified (n = 4). Scale bar: 20 µm. Data are shown as mean ± SD. *p < 0.05, **p < 0.01, ***p < 0.001

We further investigated the effects of YME1L on renal histopathological changes. The hematoxylin–eosin (H&E) staining showed that diabetes caused renal tubular cell atrophy, and tubular vacuolization, while SIRIUS RED and MASSON staining indicated an increased deposition of collagen and fiber in diabetic mice. Whereas these abnormalities were attenuated in YME1L-overexpressing mice (Fig. 2A-C). Moreover, YME1L overexpression led to the downregulation of fibrotic markers such as α-Smooth Muscle Actin (α-SMA) and fibronectin (FN), accordingly, the expression of mesenchymal cell markers (vimentin and N-cadherin) was significantly downregulated by YME1L overexpression as shown by western blotting and immunohistochemical staining (Fig. 2D-K). Altogether, this information suggests that YME1L is downregulated in response to DKD and its overexpression prevents renal injury in HFD/STZ-induced diabetic mice.

### YME1L alleviates cellular senescence in DKD mice and HK2 cells challenged by HG

**Fig. 3:**
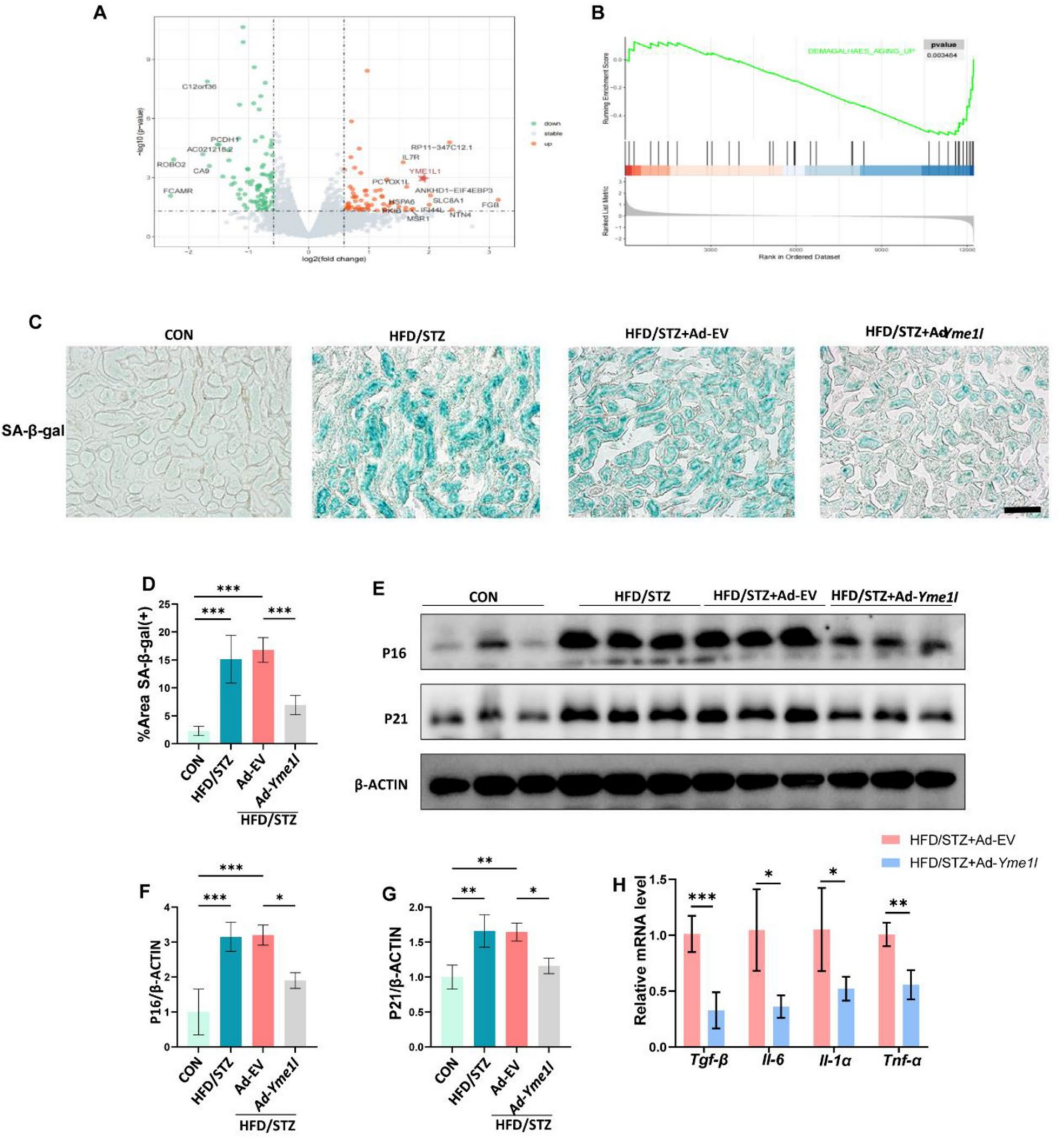
Overexpression of YME1L in the renal tubules alleviates the senescent phenotype of RTECs in HFD/STZ-induced DKD. **A** Volcano plots displayed the differentially expressed genes between Vector and YME1L overexpression HK2 cells in HG conditions. **B** GSEA revealed negative enrichment of aging up. **C** Representative SA-β-Gal-staining micrographs indicated senescent tubules with blue color, and **D** the percentage of positive tubules was quantified (n = 4). Scale bar: 100 µm. **E**–**G** Western blotting and associated quantitative analysis of kidney P16, P21 protein expression in each group (n = 4). **H** RT-PCR analysis of kidney *Tgf-β*, *Il-6*, *Il-1α*, and *Tnf-α* mRNA expression from HFD/STZ + Ad-EV and HFD/STZ + Ad-*Yme1l* mice (n = 4). Data are shown as mean ± SD. *p < 0.05, **p < 0.01, ***p < 0.001

To explore the mechanisms of YME1L ameliorating renal tubule injury, transcriptional changes in HK2 cells under HG conditions were analyzed via RNA sequencing post-transfection with YME1L-plasmid or controls. A comparison of the YME1L-overexpressed and control groups identified 165 RNA species that exhibited statistically significant changes and differential expression (75 upregulated genes and 90 downregulated genes) (Fig. 3A, Additional file 2: Table S5). GSEA indicated that genes related to aging up were significantly repressed by YME1L-overexpression (Fig. 3B). Mounting evidence suggests that cellular senescence is an important feature of aging and is associated with renal dysfunction in DKD [12]. Consistent with previous research [12], immunohistochemical staining revealed that the protein levels of P16 and P21 were significantly elevated in the renal tubules of patients with DKD compared to those of normal subjects (Additional file 1: Figure S4A-C). This finding was further supported by in vitro studies. HG induced cellular senescence phenotype in HK2 cells, cytoplasmic SA-β-Gal activity and the protein levels of P16, P21 increased significantly at 72 h in HG-treated HK2 cells (Additional file 1: Figure S4D-H). Therefore, senescent RTECs in HFD/STZ-induced DKD mice were examined to study the mechanism of YME1L in improving renal function.

In the kidneys of HFD/STZ mice, the expression of senescence-associated markers, including cytoplasmic SA-β-Gal activity and the protein levels of P16, P21 were increased, YME1L overexpression markedly decreased these effects (Fig. 3C-G). Moreover, YME1L overexpression led to reduced mRNA abundance of common SASP markers, including *Tgf-β*, *Il-6*, *IL-1α*, and *Tnf-α,* compared with control mice with diabetes (Fig. 3H). These findings suggest that YME1L may ameliorate renal tubule injury by suppressing cellular senescence and SASP.

**Fig. 4:**
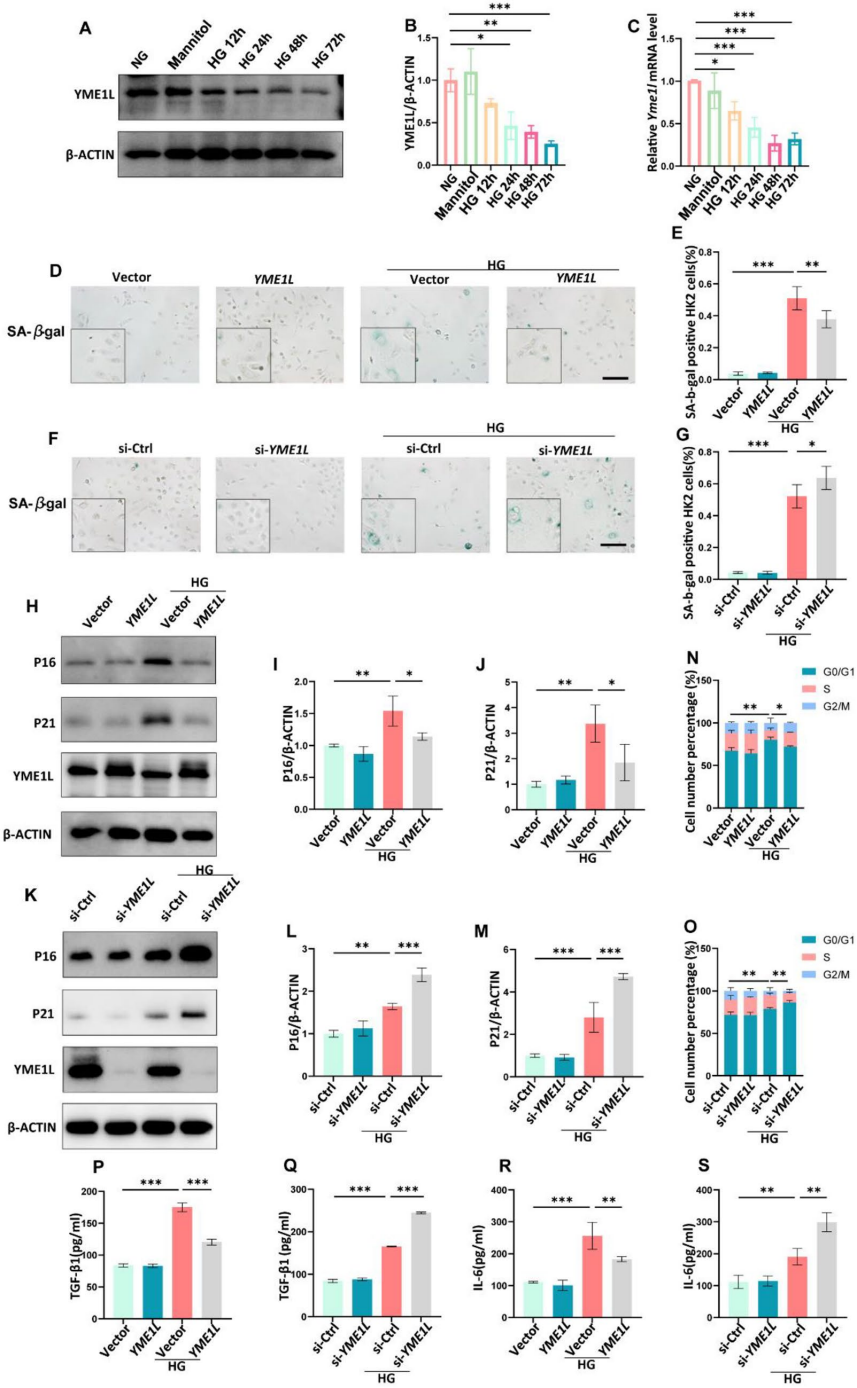
HG-induced cellular senescence is restrained by YME1L overexpression and accelerated by silencing of YME1L in HK2 cells. **A**, **B** Western blotting and associated quantitative analysis of YME1L in HK2 cells at different time points after HG treatment (n = 4). **C** RT-PCR analysis of YME1L mRNA expression in HK2 cells at different time points after HG treatment (n = 3). **D**-**G** Representative SA-β-Gal-staining micrographs of HK2 cells transfected with YME1L-plasmid, si-YME1L, and their corresponding controls on stimulation with D-glucose for 72 h, the percentage of positive cells was quantified (n = 4). Scale bar: 200 µm. **H**-**M** Western blotting and associated quantitative analysis of P16, P21 expression in HK2 cells transfected with YME1L-plasmid, si-YME1L, and their corresponding controls on stimulation with D-glucose for 72 h (n = 4). **N**, **O** The number of cells in different cell cycles detected by flow cytometry in HK2 cells transfected with YME1L-plasmid, si-YME1L, and their corresponding controls on stimulation with D-glucose for 72 h (n = 4). **P**-**S** Bar charts showed the amount of TGF-β1, IL-6 in the cellular supernatant of HK2 cells transfected with YME1L-plasmid, si-YME1L, and their corresponding controls on stimulation with D-glucose for 72 h (n = 3). Data are shown as mean ± SD. *p < 0.05, **p < 0.01, ***p < 0.001

We further explored the anti-senescence effect of YME1L in vitro. After exposure to HG, YME1L protein and mRNA levels were downregulated in HK2 cells. As an isotonic control, mannitol did not cause significant changes in YME1L expression (Fig. 4A-C). [12, 16]. Then transfection of YME1L overexpression plasmid and siRNA (Additional file 1: Figure S5A-F) was performed to determine the effect of YME1L on RTEC senescence in vitro. Consistent with our in vivo results, YME1L-overexpression significantly decreased SA-β-Gal staining positive cells (Fig. 4D, E); inhibited the protein level of P16, P21 (Fig. 4H-J); flow cytometry showed that HK2 cells were arrested at the G0/G1 phase under HG stress, while YME1L-overexpression released HK2 cells from the G0/G1 phase (Fig. 4N); YME1L-overexpression also reduced the levels of IL-6 and TGF-β1 in the cellular supernatant in the presence of HG conditions (Fig. 4P, R). However, transfection of YME1L siRNA showed the opposite results (Fig. 4F, G, K-M, O, Q, S). Taken together, these results suggest that genetic overexpression of YME1L from renal tubule cells improves RTEC senescence associated with tubular injury under diabetic circumstances.

### YME1L improves mitochondrial structure and function

**Fig. 5:**
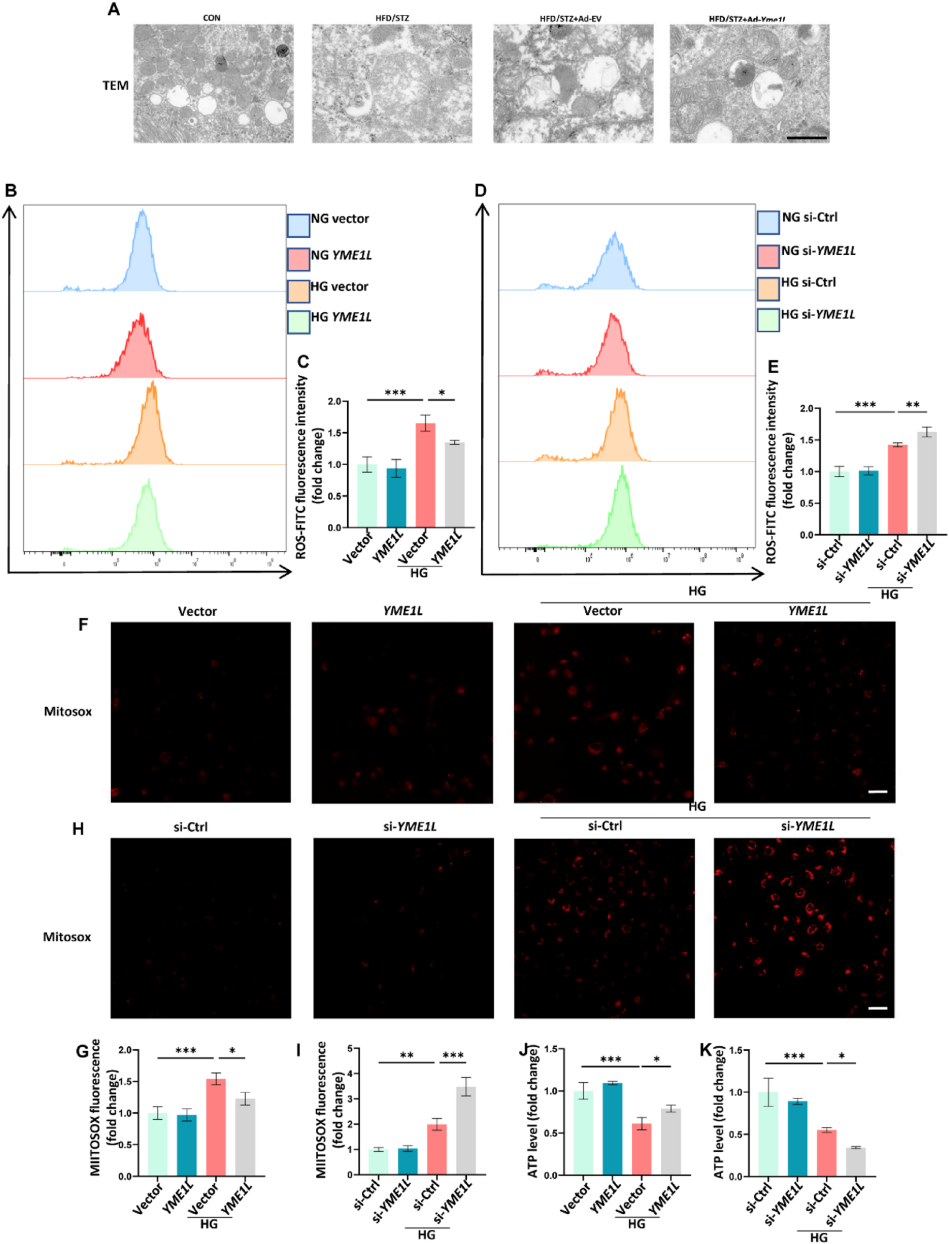
HG-induced mitochondrial dysfunction is restrained by overexpression of YME1L and accelerated by silencing of YME1L in HK2 cells. **A** TEM images of mitochondrial morphology in each group. Scale bar: 2 µm. **B**–**E** The intracellular ROS production was measured via flow cytometry in HK2 cells transfected YME1L-plasmid, si-YME1L, and their corresponding controls on stimulation with D-glucose for 48 h (n = 3). **F**–**I** Mitochondrial ROS in HK2 cells transfected YME1L-plasmid, si-YME1L, and their corresponding controls were analyzed by confocal microscopy after staining with MitoSOX. D-glucose was added 48 h before staining (n = 4). Scale bar: 50 µm. **J**, **K** ATP levels were detected via luciferase assay in HK2 cells transfected YME1L-plasmid, si-YME1L, and their corresponding controls on stimulation with D-glucose for 48 h (n = 3). Data are shown as mean ± SD. *p < 0.05, **p < 0.01, ***p < 0.001

Emerging evidence suggests that cellular senescence in RTECs is driven and maintained by mitochondrial dysfunction and oxidative stress [16]. The TEM showed that diabetes caused mitochondrial swelling, inner membrane rupture and mitochondrial crest rupture in mouse RTECs. Whereas these abnormalities were attenuated in YME1L-overexpressing mice (Fig. 5A). Next, DCFH-DA and MitoSOX Red were selected to examine intracellular and mitochondrial ROS generation, respectively. We found that HG-induced intracellular ROS production was significantly reduced in YME1L-overexpressing HK2 cells compared to control cells (Fig. 5B, C), while YME1L silencing led to further increases in intracellular ROS production (Fig. 5D, E). Additionally, MitoSOX staining revealed that the effects of YME1L on mitochondrial ROS were similar to cellular ROS in NG and HG ambiance (Fig. 5F-I). Suggested that YME1L helped buffer oxidative stress in HG conditions. Furthermore, uncontrolled oxidative stress is known to interrupt ATP production in the mitochondria [33]. In the present study, we demonstrated that YME1L overexpression was able to reverse HG-induced suppression of ATP production in HK2 cells (Fig. 5J), while YME1L silencing led to an even greater decrease in ATP production (Fig. 5K). In summary, these findings suggest that the overexpression of YME1L in HK2 cells can effectively alleviate the mitochondrial dysfunction and oxidative stress caused by HG.

### Enhanced mitophagy by YME1L protects RTECs from diabetes-induced cellular senescence

**Fig. 6:**
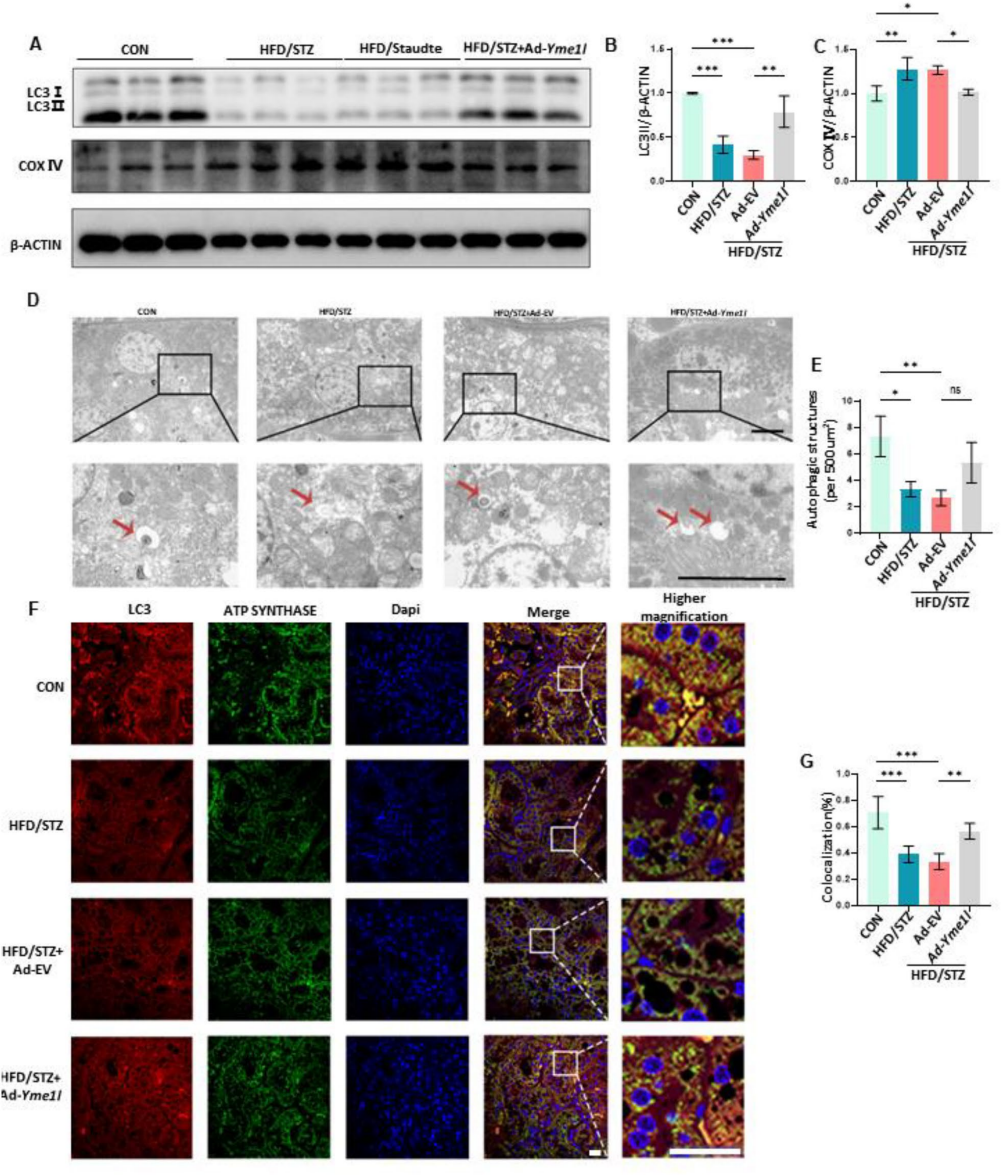
Overexpression of YME1L in the renal tubules improves mitophagy in HFD/STZ-induced DKD. **A**-**C** Western blotting and associated quantitative analysis of LC3II and COX IV in each group (n = 4). **D**, **E** Representative TEM images and statistical graphs of autophagic structures in kidney tissues in each group (n = 3). Scale bar: 5 µm. **F**, **G** Representative images and statistical graphs for immunofluorescence staining of LC3 (Red) and ATP SYNTHASE (Green) colocalization in the renal tubules (n = 5). Scale bar: 20 µm. Data are shown as mean ± SD. *p < 0.05, **p < 0.01, ***p < 0.001. ns: no statistically significant difference

Previous research has indicated that impaired mitophagy and subsequent accumulation of mitochondrial ROS play a crucial role in diabetes-associated senescence in RTECs [16]. With that in mind, we investigated whether YME1L has an impact on mitophagy. To investigate the changes in mitophagy, we measured the protein levels of LC3 II, which suggests autophagosome formation [34], as well as the mitochondrial marker protein COX IV [35], which reflects mitochondrial content. In the kidneys of HFD/STZ-induced DKD mice, western blotting analysis revealed the downregulated protein LC3II, while COX IV was increased. However, YME1L overexpression reversed these effects by increasing LC3II protein levels and reducing COX IV expression (Fig. 6A-C). Additionally, TEM images confirmed that YME1L-overexpression mice improved autophagy levels, as evidenced by decreased autophagosomes and autolysosomes in HFD/STZ mice renal tissues and upregulated by YME1L overexpression. Still, it was not statistically significant, possibly due to the small sample size (Fig. 6D, E). Subsequently, mitophagy activity was further observed via immunofluorescence, the colocalization of the mitochondrial marker ATP Synthase with LC3 was downregulated by HFD/STZ treatment and averted by YME1L overexpression (Fig. 6F, G). These data strongly suggest that YME1L overexpression enhanced mitophagy in the kidneys of HFD/STZ mice.

**Fig. 7:**
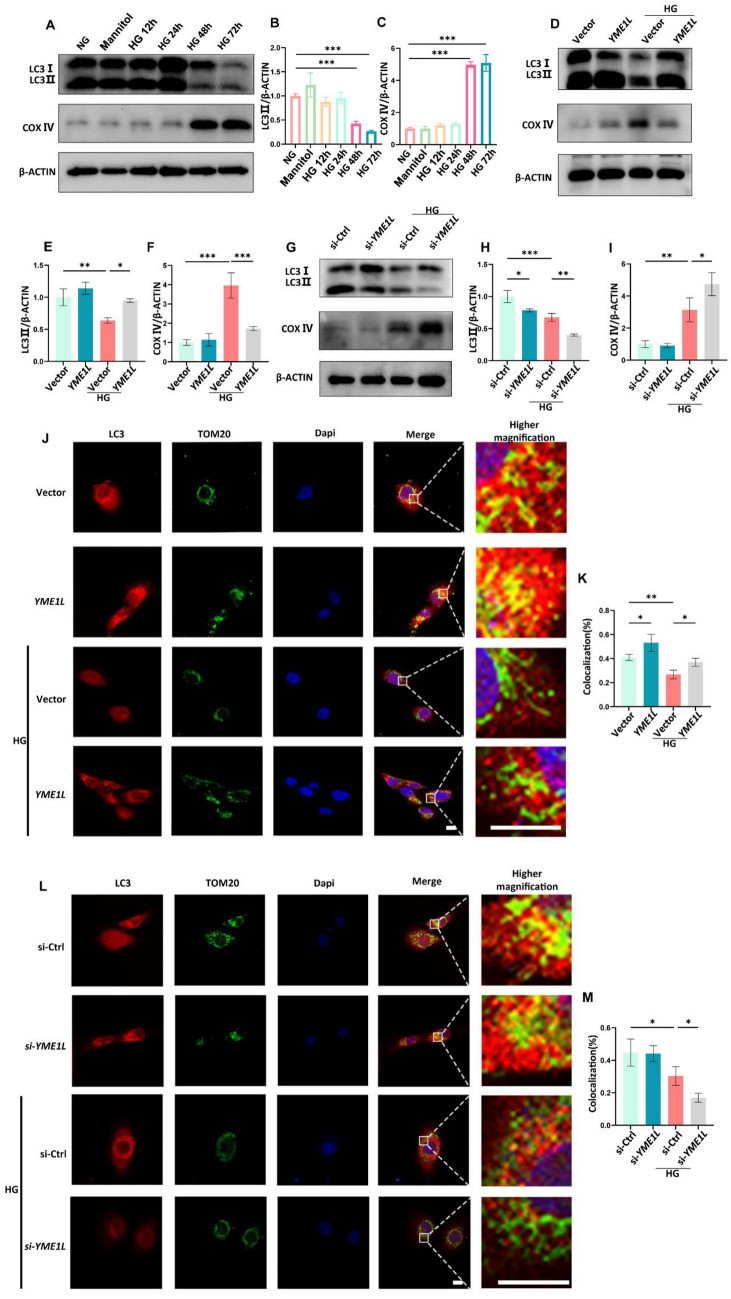
HG-mediated inhibition of mitophagy restores by overexpression of YME1L and exacerbates by silencing of YME1L in HK2 cells. **A**-**C** Western blotting and associated quantitative analysis of LC3II, COX IV in HK2 cells at different time points after HG treatment (n = 3). **D**-**I** Western blotting and associated quantitative analysis of LC3II, COX IV expression in HK2 cells transfected with YME1L-plasmid, si-YME1L, and their corresponding controls on stimulation with D-glucose for 48 h (n = 4). **J**-**M** Representative images and statistical graphs for Immunofluorescence staining of LC3 (Red) and TOM20 (Green) colocalization in HK2 cells transfected with YME1L-plasmid, si-YME1L, and their corresponding controls on stimulation with D-glucose for 48 h (n = 4). Scale bar: 10 µm. Data are shown as mean ± SD. *p < 0.05, **p < 0.01, ***p < 0.001

Next, we investigated the effect of YME1L on mitophagy in vitro. Consistent with our in vivo results, HG treatment resulted in decreased LC3II and elevated COX IV in HK2 cells (Fig. 7A-C). However, YME1L-overexpression in HK2 cells led to increased LC3II and reduced COX IV in HG conditions (Fig. 7D-F), whereas transfection of YME1L siRNA showed the opposite results (Fig. 7G–I). As YME1L is a metalloprotease, we further explored whether its regulation of LC3II and COX IV depended on its protease activity. As shown in Additional file 1: Figure S6 A-C, HK2 cells were transfected with either the wild-type (WT) YME1L plasmid or the proteolytically inactive YME1L (E543Q) plasmid. LC3II and COX IV were at similar levels under HG conditions in both WT and E543Q mutant groups, suggesting that YME1L's protease activity was independent of its regulation of LC3II and COX IV. Mitophagy activity was further observed via immunofluorescence. HG treatment disrupted the fusion between the mitochondrial marker TOM20 and LC3. Interestingly, YME1L overexpression reactivated the interaction between TOM20 and LC3 (Fig. 7J, K), which was further decreased by silencing YME1L (Fig. 7L, M). Altogether, our data indicate that mitophagy is drastically inhibited by HG and is re-activated by YME1L.

**Fig. 8:**
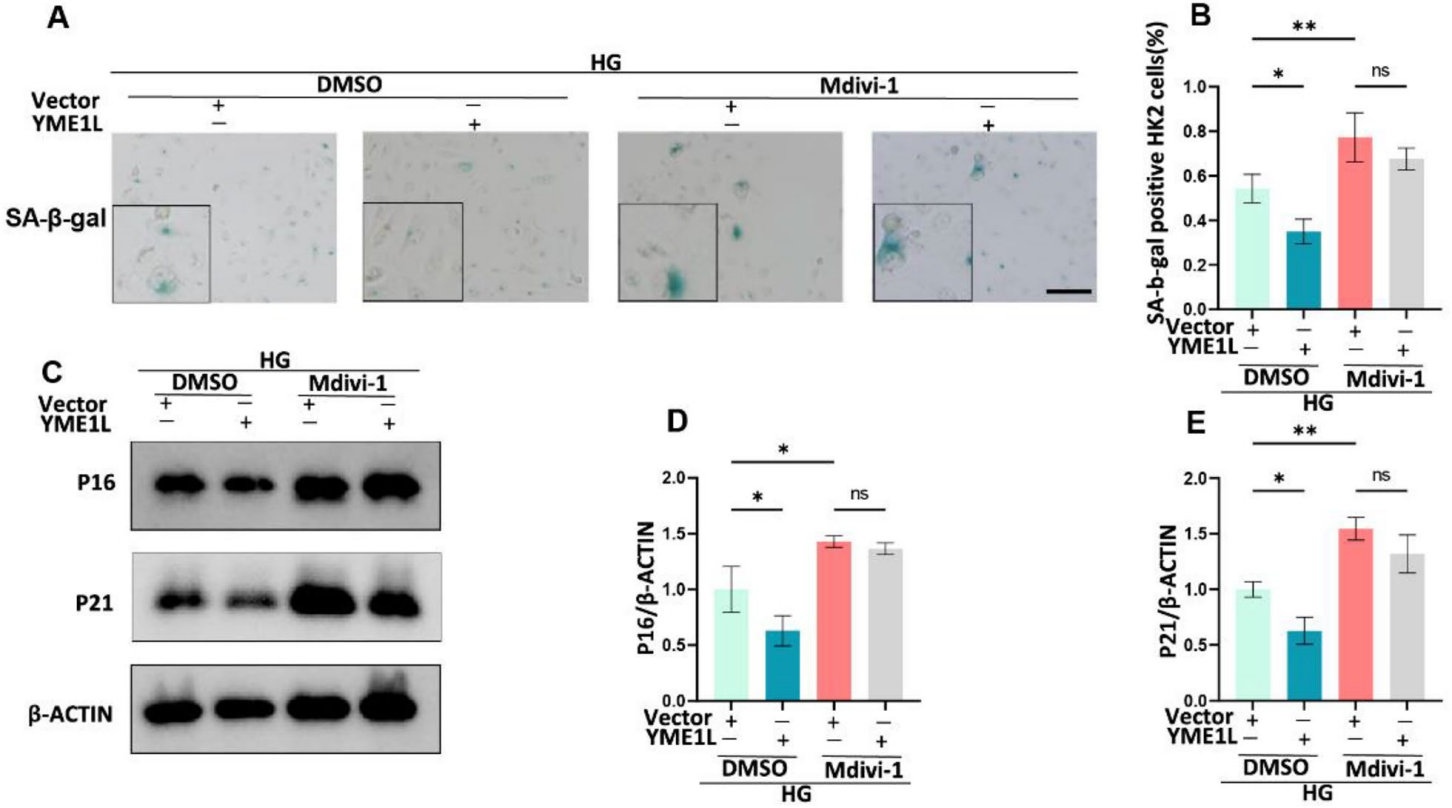
Effects of Mdivi-1 on YME1L alleviating RTEC senescence. **A**, **B** Representative SA-β-Gal-staining micrographs of HK2 cells transfected with YME1L-plasmid in HG conditions and treated with DMSO or Mdivi-1 (5 µM) for 6 h (n = 4). Scale bar: 200 µm. **C**-**E** Western blotting and associated quantitative analysis of P16, and P21 in HK2 cells transfected with YME1L-plasmid in HG conditions and treated with DMSO or Mdivi-1 (5 µM) for 6 h (n = 4). Data are shown as mean ± SD. *p < 0.05, **p < 0.01. ns: no statistically significant difference

To further validate the importance of reconstructed mitophagy in YME1L inhibition on RTEC senescence under HG ambiance, we pretreated HK2 cells with the mitophagy inhibitor Mdivi-1 [36]. We observed that administering Mdivi-1 prevented the reduction of senescence-associated markers in HK2 cells that were overexpressing YME1L. As a result, the levels of senescence-associated markers, including SA-β-Gal activity, P16, and P21, were comparable in both Mdivi-1 treated control and YME1L overexpression HK-2 cells (Fig. 8A-E). Collectively, these data strongly suggest that YME1L overexpression enhanced mitophagy in RTECs, which in turn slowed down RTEC senescence.

### The interaction of YME1L and BCL2L13 mediates the mitophagy and relieves cellular senescence in RETCs

**Fig. 9:**
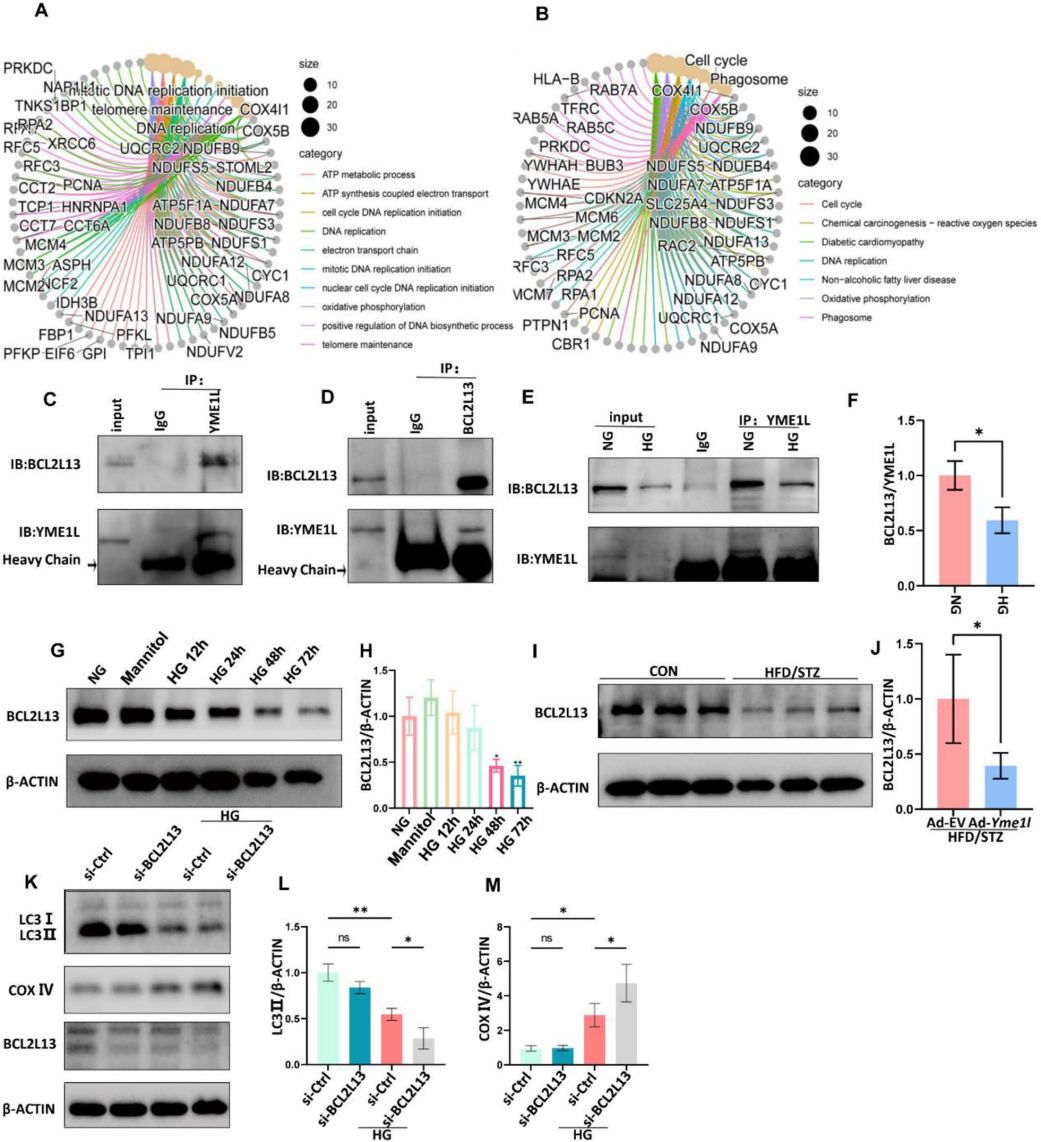
BCL2L13 was identified as an important interacting protein of YME1L by proteomics. **A** KEGG pathway analysis of YME1L-interacting proteins. **B** GO analysis of YME1L-interacting proteins. **C**, **D** Reciprocal Co-IP showing the interaction of YME1L and BCL2L13 in HK2 cells. Arrows indicate specific bands. **E**, **F** HK2 cells were incubated with HG for 48 h, and then cell lysates were immunoprecipitated with anti-YME1L, and co-precipitated BCL2L13 were detected by western blotting and the associated quantitative analysis (n = 3). **G**, **H** Western blotting and associated quantitative analysis of BCL2L13 in HK2 cells at different time points after HG treatment (n = 4). **I**, **J** Western blotting and quantitative analysis of kidney BCL2L13 expression in CON and HFD/STZ mice (n = 6). **K**-**M** Western blotting and associated quantitative analysis of LC3II, COX IV expression in HK2 cells transfected with si-BCL2L13 and si-Ctrl on stimulation with D-glucose for 48 h (n = 4). Data are shown as mean ± SD. *p < 0.05, **p < 0.01

To further investigate the mechanism by which YME1L modulates the mitophagy of RTECs under HG conditions, we performed LC–MS/MS analysis to identify YME1L-binding proteins in NG control and HG groups, revealing hundreds of potential YME1L-binding proteins from HK2 cells (Additional file 1: Figure S7A, Additional file 1: Table S6, S7). To understand the biological roles of putative targets of YME1L, we performed Kyoto Encyclopedia of Genes and Genomes (KEGG) analysis and Gene Ontology (GO) analysis using YME1L-binding partners in the NG control group. KEGG pathway analysis revealed that YME1L-interacting proteins were enriched in “Cell cycle”, “Chemical carcinogenesis—reactive oxygen species”, “Oxidative phosphorylation” and “Phagosome”. These findings suggest the regulatory role of YME1L in controlling mitochondrial function and autophagy. GO analysis suggested that YME1L-interacting proteins were involved in various biological processes, including mitochondrial function-related processes such as “ATP metabolic process”, “ATP synthesis coupled electron transport”, and “Electron transport chain”. Additionally, these proteins were also implicated in cellular senescence -related processes like “DNA replication” and “Telomere maintenance” (Fig. 9A, B). In the differentially expressed proteins between the NG control and HG groups (Additional file 1: Figure S6 B), we were specifically interested in BCL2L13, an outer mitochondrial membrane protein that functions as a crucial mitophagy receptor in mammalian cells. BCL2L13 interacts with LC3II directly to promote mitophagy through the conserved LC3-interacting region (LIR) sequence, phosphorylated BCL2L13 has a stronger ability to bind with LC3II to allow recruitment of mitophagy machinery. We validated the endogenous association of YME1L and BCL2L13 in HK2 cells (Fig. 9C,D), and found that HG treatment decreased the interaction between them (Fig. 9E, F).

We further investigated the expression of BCL2L13 in DKD. After HG exposure, the BCL2L13 protein levels were downregulated in HK2 cells (Fig. 9G, H), while the mRNA expression remained comparable (Additional file 1: Figure S8A). The expression of BCL2L13 in vivo experiment was consistent with those of the in vitro (Fig. 9I, J, Additional file 1: Figure S8B)*.* We then transfected HK2 cells with BCL2L13 siRNA (Additional file 1: Figure S9A-C) to determine the effect of its downregulation on mitophagy. Our results showed that silencing of BCL2L13 in HK2 cells reduced LC3II levels and increased COX IV expression under HG conditions (Fig. 9K-M). These results suggested that the downregulation of BCL2L13 further reduces the level of mitophagy under HG conditions, indicating that BCL2L13 is an important interacting protein of YME1L in the regulation of mitophagy in DKD.

**Fig. 10:**
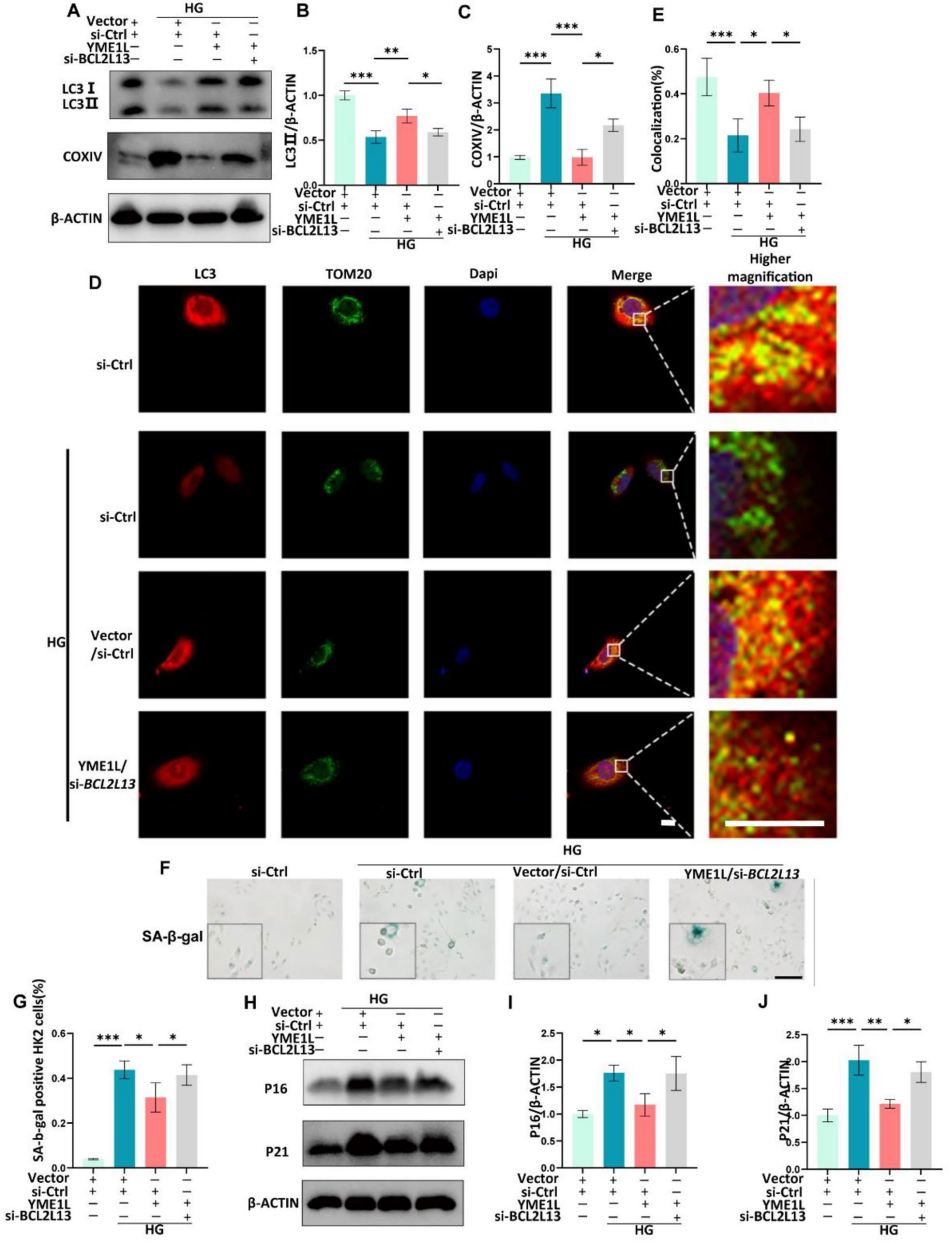
YME1L improves mitophagy and alleviates the senescent phenotype of HK2 cells in the presence of HG via BCL2L13. **A**-**C** Western blotting and associated quantitative analysis of LC3II, COX IV expression in HK2 cells transfected with YME1L plasmid, BCL2L13 si-RNA, and their controls on stimulation with D-glucose for 48 h (n = 4). **D**, **E** Immunofluorescence demonstrating LC3 (Red) and TOM20 (Green) colocalization in HK2 cells transfected with YME1L-plasmid, BCL2L13 si-RNA, and their controls on stimulation with D-glucose for 48 h (n = 4). Scale bar: 10 µm. **F**, **G** Representative SA-β-Gal-staining micrographs of HK2 cells transfected with YME1L plasmid, BCL2L13 si-RNA, and their controls on stimulation with D-glucose for 72 h, and the percentage of positive cells were quantified (n = 4). Scale bar: 200 µm. **H**-**J** Western blotting and associated quantitative analysis of P16, P21 expression in HK2 cells transfected with YME1L plasmid, BCL2L13 si-RNA and their controls on stimulation with D-glucose for 72 h (n = 4). Data are shown as mean ± SD. *p < 0.05, **p < 0.01, ***p < 0.001

To determine the role of the YME1L-BCL2L13 interaction in mitophagy regulation and senescent phenotype in HK2 cells in the presence of HG stress, YME1L plasmid, and BCL2L13 si-RNA were co-transfected in vitro. As shown in Fig. 10A-E, BCL2L13 elimination decreased the impact of YME1L on LC3II, COX IV protein levels, and LC3II colocalization with TOM20 in response to HG treatment. Moreover, the downregulation of cytoplasmic SA-β-Gal activity and the protein levels of P16, and P21 induced by YME1L overexpression was abolished by BCL2L13 knockdown (Fig. 10F-J). Together, these results strongly support the view that tubular cell YME1L enhances mitophagy and prevents cellular senescence via BCL2L13.

**Fig. 11:**
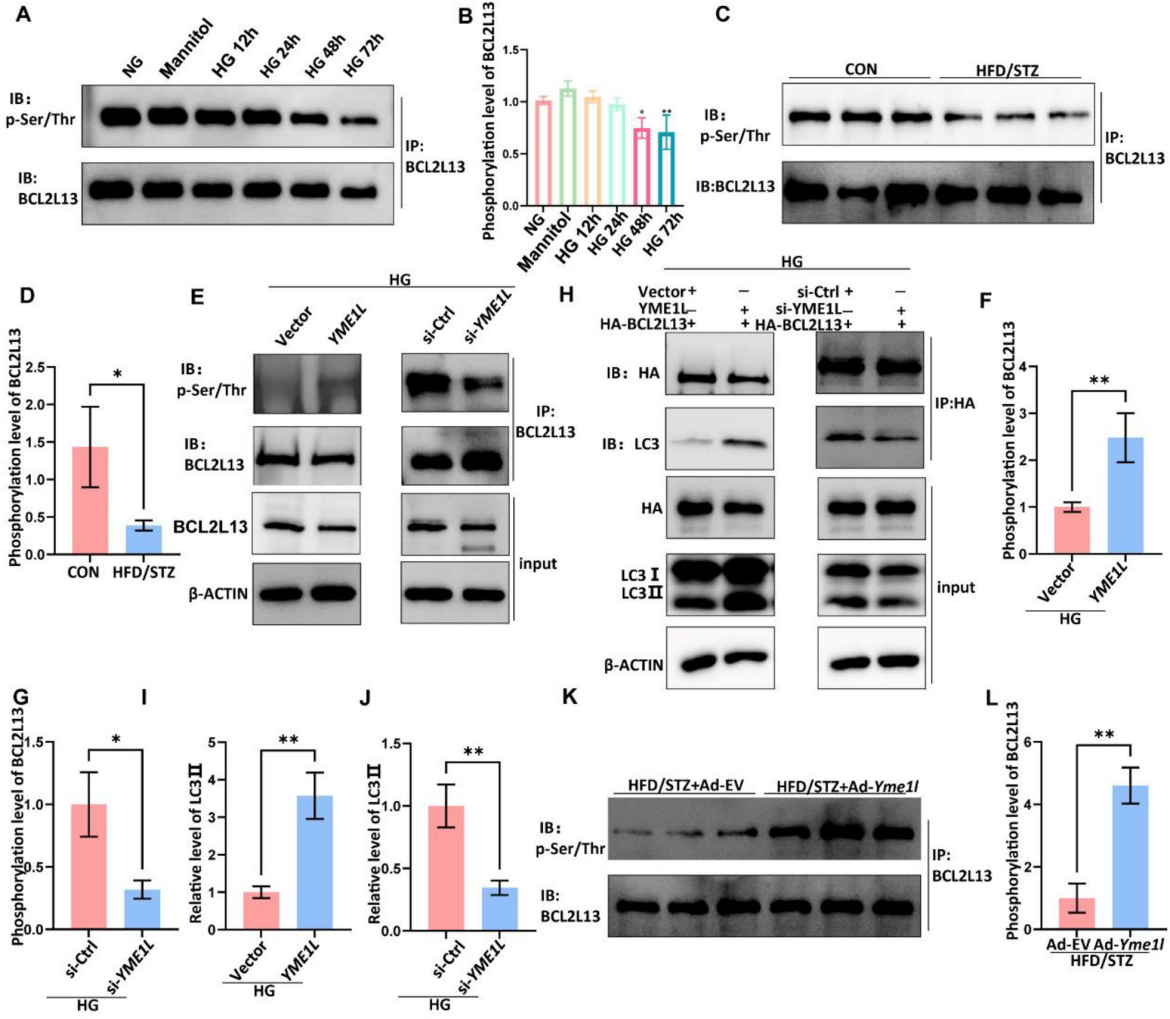
YME1L promotes the phosphorylation of BCL2L13. **A**, **B** Phosphorylation of BCL2L13 was detected by IP with anti-BCL2L13 antibody of HK2 cells treated with HG at different time points (n = 3). **C**, **D** Phosphorylation of BCL2L13 was detected by IP with anti-BCL2L13 antibody of kidney tissues from CON and HFD/STZ mice (n = 3). **E**–**G** Phosphorylation of BCL2L13 was detected by IP with anti-BCL2L13 antibody of HK2 cells transfected with YME1L plasmid, YME1L si-RNA, and their controls on stimulation with D-glucose for 48 h (n = 3). **H**-**J** HK2 transfected with HA-BCL2L13, the protein level of LC3 was detected by IP with anti-HA antibody of HK2 cells transfected with YME1L plasmid, YME1L si-RNA, and their controls on stimulation with D-glucose for 48 h (n = 3). **K**, **L** Phosphorylation of BCL2L13 was detected by IP with anti-BCL2L13 antibody of kidney tissues from HFD/STZ + Ad-EV and HFD/STZ + Ad-*Yme1l* mice (n = 3). Data are shown as mean ± SD. *p < 0.05, **p < 0.01, ***p < 0.001

We further explored the effect of YME1L on BCL2L13 expression. The protein levels of BCL2L13 did not change after transfection of the YME1L overexpression plasmid or YME1L-siRNA regardless of exposure to HG conditions (Additional file 1: Figure S10A-D). Similarly, the protein levels of BCL2L13 in kidney tissue of diabetic mice were not different from the Ad-*Yme1l* group and Ad-EV group, which is consistent with our in vitro results (Additional file 1: Figure S10E, F). It has been reported that BCL2L13 can be phosphorylated at the posttranslational level to promote mitophagy, we next explored whether YME1L regulates the phosphorylation status of BCL2L13. As shown in Fig. 11A-D, Phosphorylated BCL2L13 was downregulated in HG-treated HK2 cells and the kidneys of diabetic mice. YME1L overexpression increased BCL2L13 phosphorylation, while YME1L knockdown led to reduced phosphorylation of BCL2L13 in vitro (Fig. 11E-G). Due to Phosphorylated BCL2L13 has a stronger ability to bind LC3, thereby promoting mitophagy. We further investigated the effect of YME1L on the binding between BCL2L13 and LC3, as shown in Fig. 11H-J. Overexpression of YME1L in HK2 cells increased the binding between BCL2L13 and LC3, while YME1L knockdown weakened this binding. Finally, we also confirmed in vivo that overexpression of YME1L can increase the phosphorylation level of BCL2L13 (Fig. 11K, L). These results suggest that YME1L plays a role in promoting mitophagy by regulating the phosphorylation of BCL2L13.

## Discussion

This study establishes the crucial role of YME1L in RTECs and the regulation of DKD. We demonstrated that YME1L-overexpression alleviated the senescent phenotype of RTECs in *vitro* and in vivo. Additionally, we have found that YME1L interacted with BCL2L13 and could regulate the phosphorylation level of BCL2L13. Collectively, our study demonstrated that YME1L reverses cellular senescence to RTECs from diabetes and prevents the progression of DKD.

Prior studies have suggested that cellular senescence is involved in age-related and senescence-associated disorders, including insulin resistance and many common complications or comorbidities of diabetes. These comorbidities encompass arteriosclerosis [37], osteoporosis [38], hepatic steatosis [39], and neurodegenerative diseases [40]. P16 and P21 act as cyclin-dependent kinase inhibitors (CKIs) [41], thereby impeding or slowing down the cell cycle and facilitating cellular senescence. Therefore, we chose these two markers to reflect cellular senescence. Previous research utilizing whole-body *p21* knockout mice has already unveiled the significance of P21 in DKD. The absence of *p21* offers protection for the glomeruli and tubules in diabetic mice.[42]. Furthermore, it has been suggested that P21 in RTECs is a major factor contributing to high glucose memory in DKD [43]. In addition, knocking out *p16* in HK2 cells has been shown to significantly downregulate the expression levels of fibrosis-related genes [12], clearing senescent cells and down-regulating SASP can improve renal tubule interstitial injury and reduce renal inflammation in diabetes [13]. Clinical studies have confirmed the crucial role of cellular senescence in the progression of DKD. Studies have demonstrated that senescent RTECs increase gradually as DKD progresses in human renal tissue specimens. Furthermore, there is a close correlation between the accumulation of senescent RTECs and the deterioration of renal function in patients with DKD [44]. The above evidence indicates the important role of cellular senescence in the progression of DKD. Clinical studies on anti- senescence treatments have been conducted [45], but further research is still needed to explore the specific role of cellular senescence in the progression of DKD, as well as the effectiveness and safety of anti- senescence therapies in this context. Our findings suggest that YME1L may improve renal function by regulating cellular senescence and targeting YME1L could be a promising therapeutic strategy for preventing and treating renal dysfunction in DKD.

In recent years, evidence has been mounting that mitochondrial dysfunction is a major contributor to cellular senescence, dysfunctional mitochondria accumulate in senescent cells, leading to decreased oxidative phosphorylation efficiency and increased ROS production [46–48]. In the present study, TEM indicated that YME1L overexpression could improve mitochondrial structure damage of renal cortex in diabetic mice as well as maintained ATP levels and buffered oxidative stress of HK2 cells in HG conditions. This was in accordance with a previously identified role for YME1L in promoting mitochondrial function [24, 49]. The theory of Senescence-Associated Mitochondrial Dysfunction (SAMD) proposes that well-orchestrated mitophagy, the process of removing damaged mitochondria, is crucial for preventing cellular senescence [17]. Given this, we focused on the role of YME1L in regulating mitophagy and used the mitophagy inhibitor Mdivi-1 to verify the protective role of YME1L on cellular senescence. Decreased mitophagy activity has been acknowledged as an etiology for cellular senescence in several diseases in addition to DKD, such as senile osteoporosis [50], chronic obstructive pulmonary disease [51], and ataxia telangiectasia [52]. In the present study, YME1L-mediated mitophagy is functionally linked with cellular senescence and has a central role in DKD.

To elucidate the molecular mechanism by which YME1L deficiency affects DKD, we performed LC–MS/MS and found that BCL2L13 interacted with YME1L in HK2 cells. BCL2L13 is ubiquitously expressed and localized in the outer mitochondrial membrane and contains a C-terminal single transmembrane domain [53]; it was reported to be a functional mammalian homolog of ATG32 in yeast [53, 54]. Although the regulatory mechanisms underlying BCL2L13-mediated mitophagy remain largely unknown, it has been reported that BCL2L13 could be a crucial mitophagy receptor for binding with LC3 to promote mitophagy. In this study, we demonstrated that YME1L promoted mitophagy by interacting with BCL2L13 and promoting its phosphorylation levels, which in turn alleviates cellular senescence.

## Conclusions

**Fig. 12:**
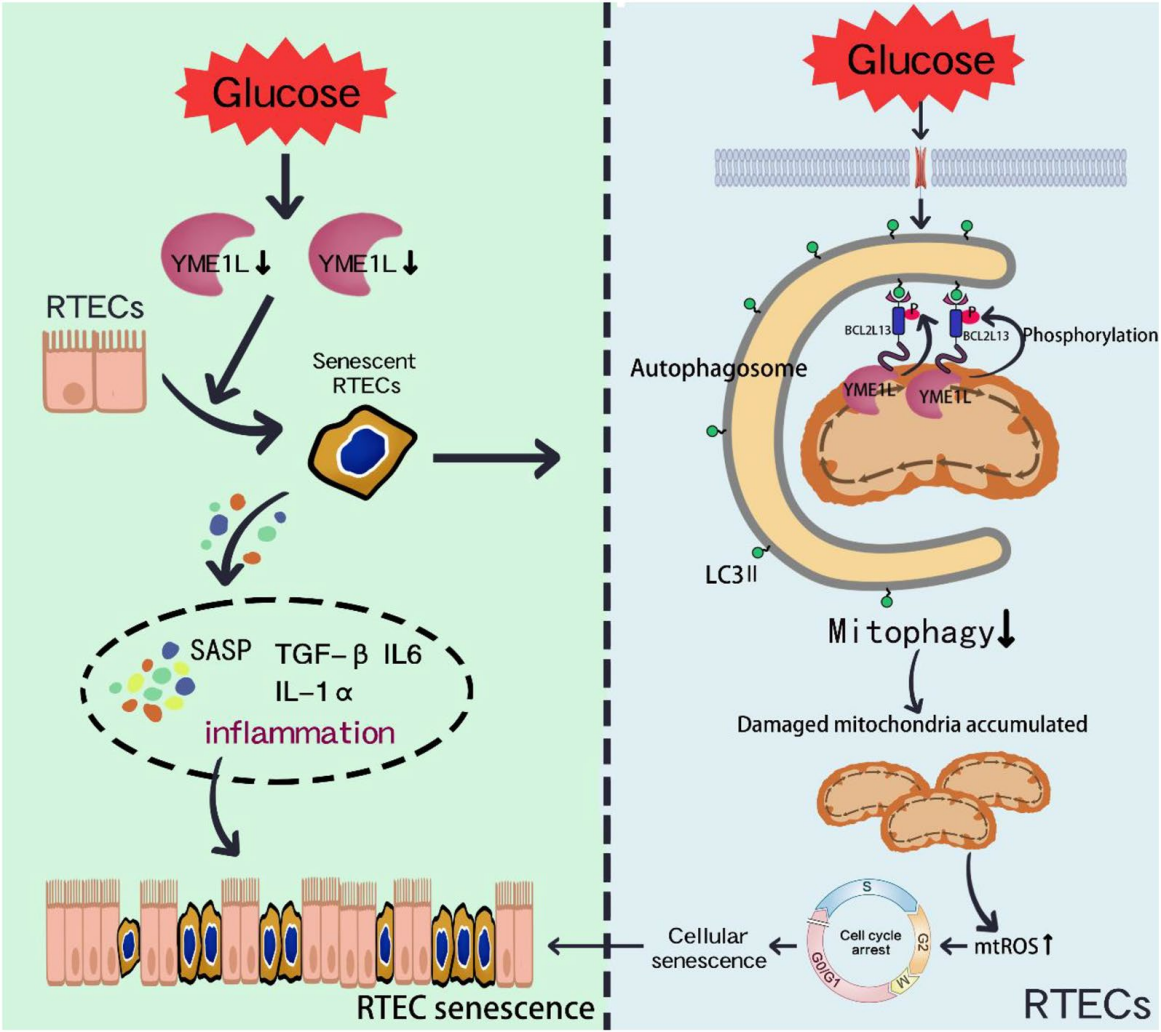
Scheme of the molecular mechanisms underlying YME1L regulates the senescent phenotype and mitophagy of RTECs. The downregulation of YME1L expression under HG ambiance disrupts mitophagy by reducing the phosphorylation of BCL2L13 and the combination between BCL2L13 and LC3, which in turn exacerbate cellular senescence of RTECs

In summary, we found that YME1L may have a significant role in mitigating the senescent phenotype of RTECs and improving renal dysfunction in DKD. By interacting with BCL2L13 and increasing its phosphorylation levels, thereby promoting mitophagy and alleviating RTEC senescence (Fig. 12). These results suggest that YME1L could serve as a potential target for the elimination of senescent cells to prevent DKD progression.

### Supplementary Information

**Additional file 1: Table S1.** The clinical characteristics of normal control subjects, EDKD patients, LDKD patients and LN patients. **Table S2.** Target sequences of siRNA. **Table S3.** Primary antibodies used for western blotting, immunoprecipitation, immunohistochemistry and immunofluorescence. **Table S4.** Sequences of primers used in RT-PCR analysis. **Figure. S1.** Expression pattern of YME1L in kidneys from normal control Subjects and LN patients. (A) Representative immunohistochemical staining of YME1L in renal tissues from normal control subjects (CON) and LN patients and (B) the percentage of YME1L expression area was quantified (n = 5-10). Scale bar: 40 µm. Data are shown as mean ± SD. ns: no statistically significant difference.. **Figure. S2.** YME1L expression in the renal tissue of mouse after treatment with Ad-*Yme1l*. (A) Representative immunohistochemical staining of HA tag in mouse kidney in CON, Ad-EV and Ad-*Yme1l* mice (n = 3). Scale bar: 20 µm. (B) Representative immunohistochemical staining of YME1L in mouse kidneys in each group, and (C) the percentage of YME1L expression area was quantified (n = 3). Scale bar: 20 µm. (D, E) Western blotting and associated quantitative analysis of kidney YME1L expression in HFD/STZ + Ad-EV and HFD/STZ + Ad-*Yme1l* mice (n = 6). (F) RT-PCR analysis of kidney *Yme1l* mRNA expression in HFD/STZ + Ad-EV and HFD/STZ + Ad-*Yme1l* mice (n = 4). Data are shown as mean ± SD. *p < 0.05, **p < 0.01. ***p < 0.001. **Figure S3.** Biochemical indicators at the termination of the study from each group. (A) UACR, (B) Blood glucose, (C) Serum urea, (D) Serum creatinine, (E) Total triglyceride, (F) Total cholesterol, (G) Low-density lipoprotein in each group (n = 8-10). Data are shown as mean ± SD. *p < 0.05, **p < 0.01, ***p < 0.001, ns: no statistically significant difference. **Figure. S4.** Diabetes-induced cellular senescence of RTECs. (A) Representative immunohistochemical staining of P16, P21 in renal tissues from normal subjects, DKD patients and (B, C) the percentage of P16, P21 expression area was quantified (n = 5). Scale bar: 40 µm. (D) Representative SA-β-Gal-staining micrographs of HK2 cells at different time points after HG treatment, and (E) the percentage of positive cells were quantified (n = 4). Scale bar: 200 µm. (F-H) Western blotting and associated quantitative analysis of P16, and P21 at different time points after HG treatment (n = 3). Data are shown as mean ± SD. *p < 0.05, ***p < 0.001. **Figure. S5.** Effects of YME1L plasmid and siRNA transfection on the expression of YME1L in HK2 cells. (A-D) The protein level of YME1L was measured by western blotting and associated quantitative analysis in HK2 cells transfected with YME1L-plasmid, si-YME1L (n = 3). (E, F) RT-PCR analysis of YME1L mRNA expression in HK2 cells transfected with YME1L-plasmid, si-YME1L (n = 3). Data are shown as mean ± SD. ***p < 0.001. **Figure. S6.** Effects of YME1L (WT)-plasmid or YME1L (E543Q) -plasmid transfection on the expression of LC3II and COX IV in HK2 cells. (A-C) Western blotting and associated quantitative analysis of LC3II and COX IV in HK2 cells transfected with YME1L (WT)-plasmid or YME1L (E543Q) -plasmid and their corresponding controls on stimulation with D-glucose for 48 h (n = 4). Data are shown as mean ± SD.*p < 0.05, **p < 0.01, ***p < 0.001. ns: no statistically significant difference. **Figure. S7.** Proteomics identification, functional analysis, and validation of YME1L-interacting proteins in HK2 cells. (A) IP assay was carried out using YME1L antibody or IgG (negative control antibody). Samples were electrophoresed and silver stained. (B) Venn diagram indicating YME1L-interacting proteins in NG control and HG groups. **Figure. S8.** Effects of HG on transcription levels of BCL2L13 expression. (A) RT-PCR analysis of *BCL2L13* mRNA expression in HK2 cells at different time points after HG treatment (n = 3). (B) RT-PCR analysis of kidney *Bcl2l13* mRNA expression in CON and HFD/STZ mice (n = 4). Data are shown as mean ± SD. ns: no statistically significant difference. **Figure. S9.** Effect of BCL2L13 siRNA transfection on the expression of BCL2L13 in HK2 cells. (A, B) The protein level of BCL2L13 was measured by western blotting and associated quantitative analysis in HK2 cells transfected with si-BCL2L13 (n = 3). (C) RT-PCR analysis of BCL2L13 mRNA expression in HK2 cells transfected with si-BCL2L13 (n = 3). Data are shown as mean ± SD. ***p < 0.001. **Figure. S10.** The effect of YME1L on the protein level and transcriptional level of BCL2L13. (A-D) Western blotting and associated quantitative analysis of BCL2L13 expression in HK2 cells transfected with YME1L-plasmid, si-YME1L, and their corresponding controls on stimulation with D-glucose (30 mmol/L) for 48 h (n = 3). (E, F) Western blotting and associated quantitative analysis of BCL2L13 expression in HFD/STZ + Ad-EV and HFD/STZ + Ad-*Yme1l* mice (n = 3). Data are shown as mean ± SD. *p < 0.05, **p < 0.01. ns: no statistically significant difference.

**Additional file 2.** RNA-sequencing data after overexpression of YME1L.

**Additional file 3.** **Table S6.** YME1L interacting proteins identified by proteomics in NG condition.

**Additional file 4. Table S7.** YME1L interacting proteins identified by proteomics in HG condition.

## Data Availability

The analysis data used to support the findings of this study are available from the corresponding author upon request.

## Abbreviations

AAA: ATPases associated with diverse cellular activities
ACEi: Angiotensin-converting enzyme inhibitors
Ad: Adeno-associated virus 9
Ad-EV: Empty adenoviral vectors carrying HA
Ad-Yme1l: Adeno-associated virus 9 carrying CMV-enhancer-Ksp-cadherin-promoter-Yme1l-HA
ARB: Angiotensin receptor blocker
BCL2L13: BCL2 like 13
BUN: Blood urea nitrogen
CMV-Ksp: Cytomegalovirus (CMV)-enhancer-Ksp-cadherin-promoter
Co-IP: Co-immunoprecipitation
CON: Control mice
DPP-4: Dipeptidyl peptidase-4
DKD: Diabetic kidney disease
EMT: Epithelial-mesenchymal transition
ESRD: End-stage renal disease
GO: Gene Ontology
HFD: High-fat diet
HFD/STZ: High-fat diet/Streptozotocin
HG: High-glucose medium
IP: Immunoprecipitation
KEGG: Kyoto Encyclopedia of Genes and Genomes
mtROS: Mitochondrial reactive oxygen species
NG: Normal-glucose medium
rAAV 2/9: Recombinant adeno-associated virus 2/9
ROS: Reactive oxygen species
RTECs: Renal tubular epithelial cells
SASP: Senescence-associated secretory phenotype
SA-β-Gal: Senescence-associated β-galactosidase
SGLT2: Sodium-glucose co-transporter-2
Si-BCL2L13: BCL2L13-specific small interfering RNA
Si-Ctrl: Control small interfering RNA
Si-YME1L: YME1L-specific small interfering RNA
STZ: Streptozotocin
TEM: Transmission electron microscopy
TIF: Tubulointerstitial fibrosis
UACR: Urinary albumin-to-creatinine ratio
Vector: Control vector plasmids
WT: Wild type
YME1L: Yeast mitochondrial escape 1-like 1
α-SMA: α-Smooth Muscle Actin
