# Supplementary material for: YME1L-mediated mitophagy protects renal tubular cells against cellular senescence under diabetic conditions
Source: Biol Res. 2024 Mar 17;57:10. doi: 10.1186/s40659-024-00487-0 (PMC10946153; doi:10.1186/s40659-024-00487-0)
Supplement: Supplementary file 1 — Additional file 1: Table S1. The clinical characteristics of normal control subjects, EDKD patients, LDKD patients and LN patients. Table S2. Target sequences of siRNA. Table S3. Primary antibodies used for western blotting, immunoprecipitation, immunohistochemistry and immunofluorescence. Table S4. Sequences of primers used in RT-PCR analysis. Figure. S1. Expression pattern of YME1L in kidneys from normal control Subjects and LN patients. (A) Representative immunohistochemical staining of YME1L in renal tissues from normal control subjects (CON) and LN patients and (B) the percentage of YME1L expression area was quantified (n = 5-10). Scale bar: 40 µm. Data are shown as mean ± SD. ns: no statistically significant difference.. Figure. S2. YME1L expression in the renal tissue of mouse after treatment with Ad-Yme1l. (A) Representative immunohistochemical staining of HA tag in mouse kidney in CON, Ad-EV and Ad-Yme1l mice (n = 3). Scale bar: 20 µm. (B) Representative immunohistochemical staining of YME1L in mouse kidneys in each group, and (C) the percentage of YME1L expression area was quantified (n = 3). Scale bar: 20 µm. (D, E) Western blotting and associated quantitative analysis of kidney YME1L expression in HFD/STZ + Ad-EV and HFD/STZ + Ad-Yme1l mice (n = 6). (F) RT-PCR analysis of kidney Yme1l mRNA expression in HFD/STZ + Ad-EV and HFD/STZ + Ad-Yme1l mice (n = 4). Data are shown as mean ± SD. *p < 0.05, **p < 0.01. ***p < 0.001. Figure S3. Biochemical indicators at the termination of the study from each group. (A) UACR, (B) Blood glucose, (C) Serum urea, (D) Serum creatinine, (E) Total triglyceride, (F) Total cholesterol, (G) Low-density lipoprotein in each group (n = 8-10). Data are shown as mean ± SD. *p < 0.05, **p < 0.01, ***p < 0.001, ns: no statistically significant difference. Figure. S4. Diabetes-induced cellular senescence of RTECs. (A) Representative immunohistochemical staining of P16, P21 in renal tissues from normal subjects, DKD patients and (B, C) [file 40659_2024_487_MOESM1_ESM.docx]

**Supplementary Materials**

**Table S1. The clinical characteristics of normal control subjects, EDKD patients, LDKD patients and LN patients.**

| Characteristics | CON (n=6) | EDKD (n=7) | LDKD (n=11) | LN (n=10) |
| --- | --- | --- | --- | --- |
| Age(yr) | 47.5±15.19 | 59.29±10.5 | 55.33±6.1 | 40.3±14.89 |
| HbA1c  (% (mmol/mol)) | NA | 7.73±1.26 | 8.93±3 | 5.53±0.23 |
| UACR (mg/g) | NA | 732.54±394.02 | 3944.96±3768.54 | 3243.97±3872.81 |
| Serum creatinine (µmol/l) | 70.58±21.59 | 154.62±62.62 | 211.27±170.76 | 106.94±94.7 |

**Table S2. Target sequences of siRNA**

| Gene | Target sequence |
| --- | --- |
| si*YME1L*-1 | GGAGGAAGCTAAACAAGAA |
| si*YME1L*-2 | CATGCAAGTTACAGTTCCA |
| si*YME1L*-3 | GCATCGAGATGTAGTTCCT |
| si*BCL2L13*-1 | GCTGCAAGAGCAACATCTT |
| si*BCL2L13*-2 | CCCTGGAATCACTGCAGAA |
| si*BCL2L13*-3 | GCAGCAACAACTGAACCTA |

**Table S3. Primary antibodies used for western blotting, immunoprecipitation, immunohistochemistry and immunofluorescence**

| Antibody | Target Species | Working dilutions | Catalog NO. | Supplier |
| --- | --- | --- | --- | --- |
| Anti - Phospho - (Ser/Thr) Phe | Rabbit | WB:1/1000 | ab17464 | Abcam, Cambridge, UK |
| ATP SYNTHASE | Mouse | IF:1/200 | 66037-1-Ig | Proteintech, Wuhan, China |
| BCL2L13 | Rabbit | WB:1/1000  IP:6ug | 16612-1-AP | Proteintech, Wuhan, China |
| CoraLite488 - conjugated Goat Anti-Mouse IgG (H+L) | Mouse | IF:1/400 | SA00013-1 | Proteintech, Wuhan, China |
| CoraLite594 – conjugated Goat Anti-Rabbit IgG (H+L) | Rabbit | IF:1/400 | SA00013-4 | Proteintech, Wuhan, China |
| FIBRONECTIN | Rabbit | WB:1/2000  IHC:1/100 | 15613-1-AP | Proteintech, Wuhan, China |
| HA | Rabbit | WB:1/5000  IP:6ug | 51064-2-AP | Proteintech, Wuhan, China |
| LC3 | Rabbit | WB:1/1000  IF:1/200 | 14600-1-AP | Proteintech, Wuhan, China |
| N-cadherin | Rabbit | WB:1/1000 | 22018-1-AP | Proteintech, Wuhan, China |
| P16 | Rabbit | WB:1/800 | A0262 | ABclonal, Wuhan, China |
| P21 | Rabbit | WB:1/800 | A19094 | ABclonal, Wuhan, China |
| P21 | Rabbit | WB:1/1000 | 2947T | Cell Signaling, Danvers, MA, USA |
| TOM20 | Mouse | IF:1/200 | 66777-1-Ig | Proteintech, Wuhan, China |
| Vimentin | Rabbit | WB:1/1000 | 10366-1-AP | Proteintech, Wuhan, China |
| YME1L | Rabbit | WB:1/1000  IP:6ug  IHC:1/100 | 11510-1-AP | Proteintech, Wuhan, China |
| α-Smooth Muscle Actin | Rabbit | WB:1/1000  IHC:1/100 | 14395-1-AP | Proteintech, Wuhan, China |
| β-ACTIN | Mouse | WB:1/10000 | 66009-1-Ig | Proteintech, Wuhan, China |

**Table S4. Sequences of primers used in RT-PCR analysis**

| Gene | Forward primer | Reverse primer |
| --- | --- | --- |
| H-*BCL2L13* | TACATCCTGCCCAGCGACAA | GCAGGCTTTCTGTGTGCCAA |
| H-*YME1L* | CCCATGTCTCTGCACAATCC | ACCCCTTCACGAATGATGG |
| H-β-*ACTIN* | CCCTGGACTTCGAGCAAGAGAT | GTTTTCTGCGCAAGTTAGG |
| M-*Tgf-β* | TTGCTTCAGCTCCACAGAGA | GGTCCCAGACAGAAGTTGGC |
| M-*Il6* | TGCCTTCTTGGGACTGATGC | GCCTCCGACTTGTGAAGTGG |
| M-IL-1α | CTCTAGAGCACCATGCTACAGAC | TGGAATCCAGGGGAAACACTG |
| M-Tnf-α | AACTTCGGGGTGATCGGTCC | TGGGGGCTGGGTAGAGAATG |
| M-*Yme1l* | CACCAGGAACAGGGAAGACG | TTTCTGATCCTGCTGGCTCC |
| M-*β-Actin* | TATGCTCTCCCTCACGCCAT | GCACGATTTCCCTCTCAGCT |


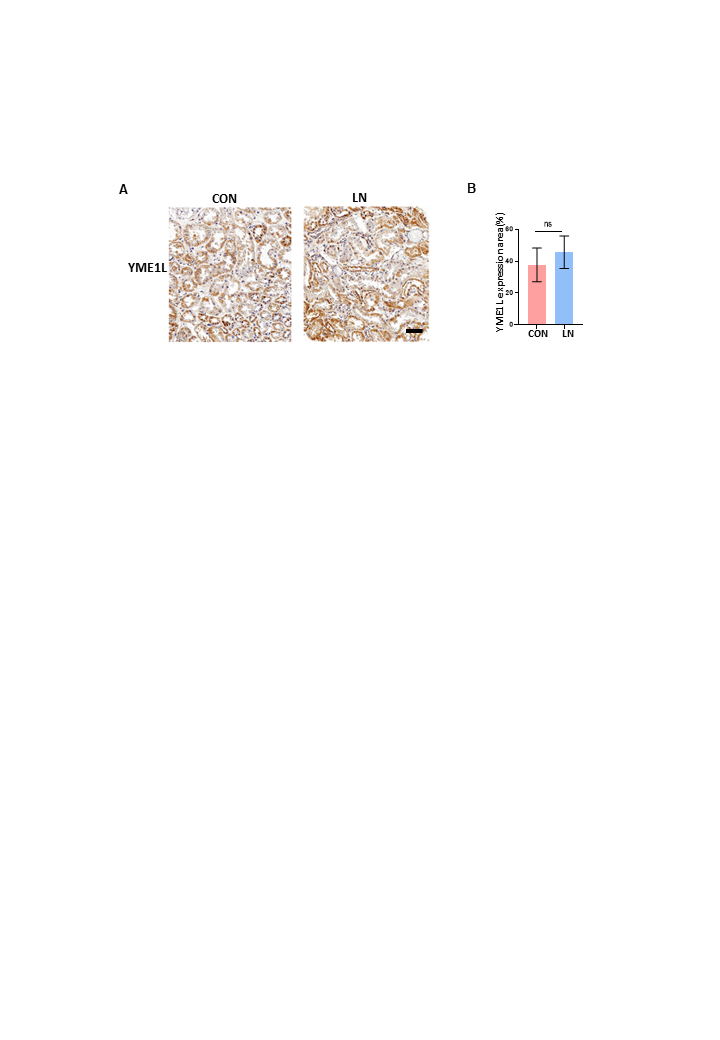


**Figure. S1 Expression pattern of YME1L in kidneys from normal control**

**Subjects and LN patients. (A) Representative immunohistochemical staining of YME1L in renal tissues from normal control subjects (CON) and LN patients and (B) the percentage of YME1L expression area was quantified (n = 5-10). Scale bar: 40 µm. Data are shown as mean ± SD. ns: no statistically significant difference.**


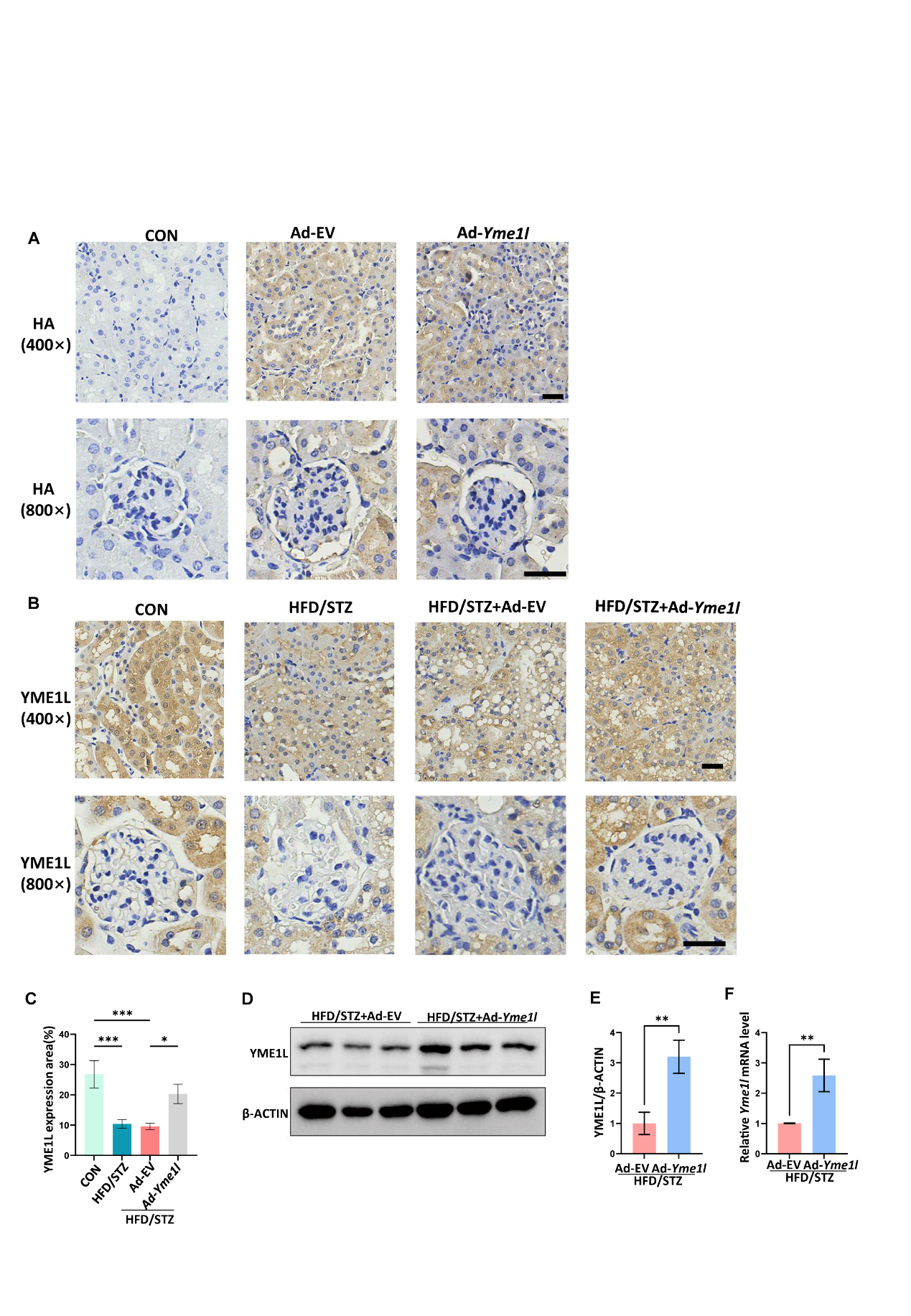


**Figure. S2 YME1L expression in the renal tissue of mouse after treatment with Ad-*Yme1l*. (A) Representative immunohistochemical staining of HA tag in mouse kidney in CON, Ad-EV and Ad-*Yme1l* mice (n = 3). Scale bar: 20 µm. (B) Representative immunohistochemical staining of YME1L in mouse kidneys in each group, and (C) the percentage of YME1L expression area was quantified (n = 3). Scale bar: 20 µm. (D, E) Western blotting and associated quantitative analysis of kidney YME1L expression in HFD/STZ+Ad-EV and HFD/STZ+Ad-*Yme1l* mice (n = 6). (F) RT-PCR analysis of kidney *Yme1l* mRNA expression in HFD/STZ+Ad-EV and HFD/STZ+Ad-*Yme1l* mice (n = 4). Data are shown as mean ± SD. **p*<0.05, ***p* < 0.01. ****p*<0.001.**


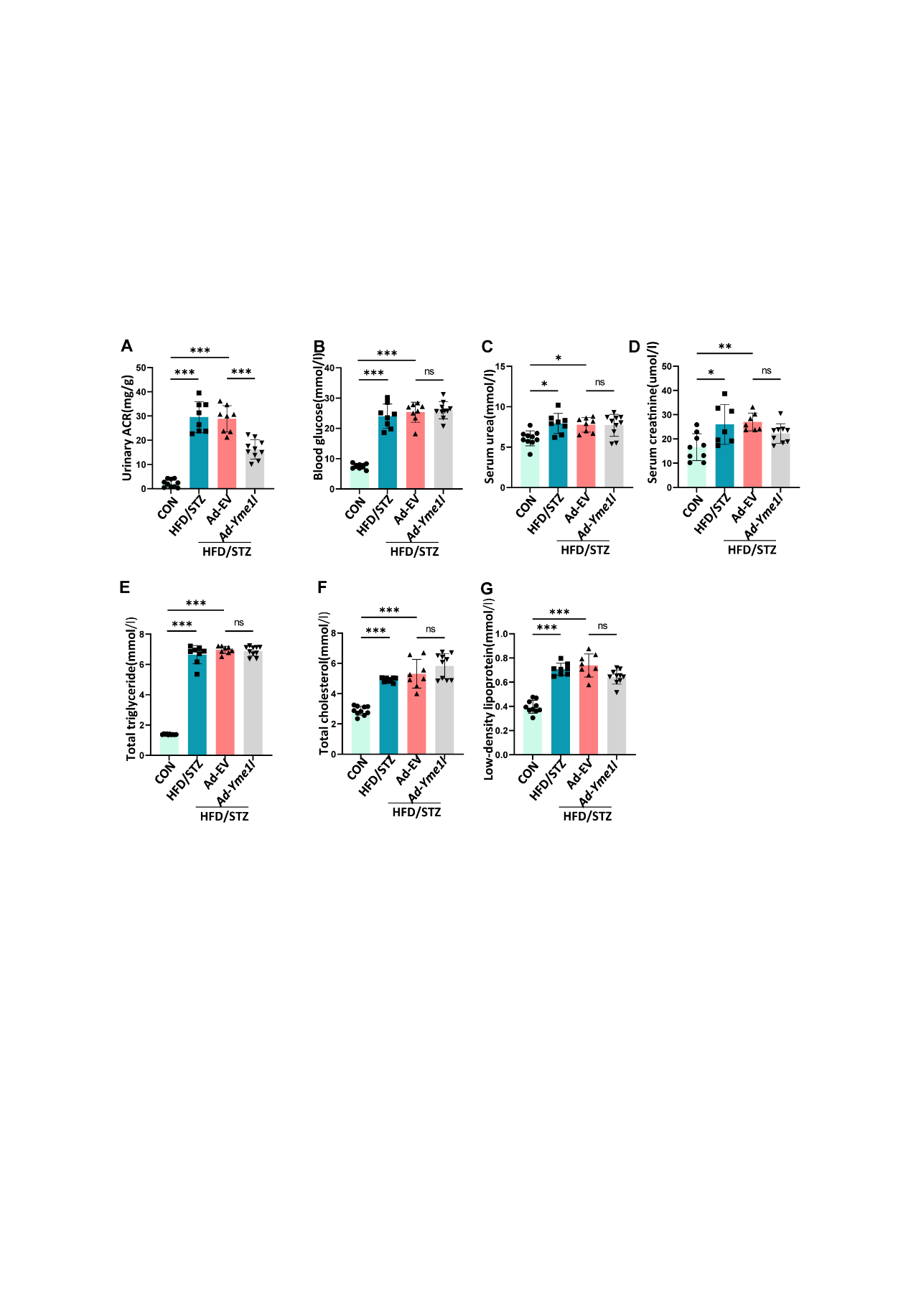


**Figure S3. Biochemical indicators at the termination of the study from each group. (A) UACR, (B) Blood glucose, (C) Serum urea, (D) Serum creatinine, (E)** **Total triglyceride, (F) Total cholesterol, (G) Low-density lipoprotein in each group (n =8-10). Data are shown as mean ± SD. **p*<0.05, ***p*<0.01, ****p*<0.001, ns: no statistically significant difference.**


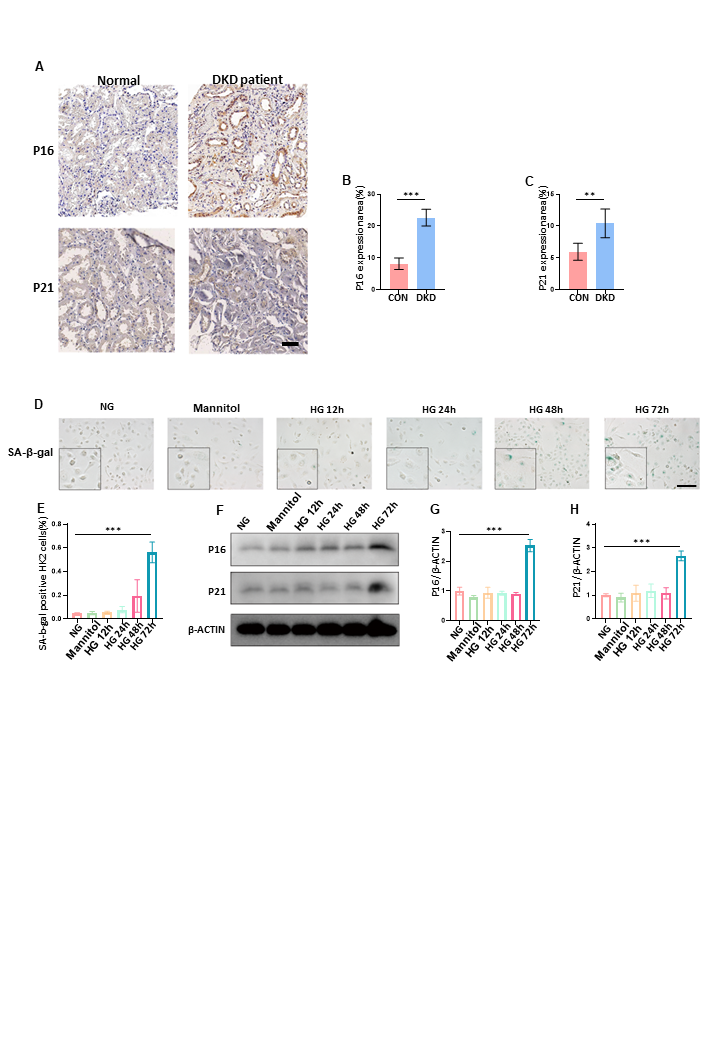


**Figure. S4 Diabetes-induced cellular senescence of RTECs. (A) Representative immunohistochemical staining of P16, P21 in renal tissues from normal subjects, DKD patients and (B, C) the percentage of P16, P21 expression area was quantified (n = 5). Scale bar: 40 µm. (D) Representative SA-β-Gal-staining micrographs of HK2 cells at different time points after HG treatment, and (E) the percentage of positive cells were quantified (n = 4). Scale bar: 200 µm. (F-H) Western blotting and associated quantitative analysis of P16, and P21 at different time points after HG treatment (n = 3). Data are shown as mean ± SD. **p*<0.05, ****p*<0.001.**

**
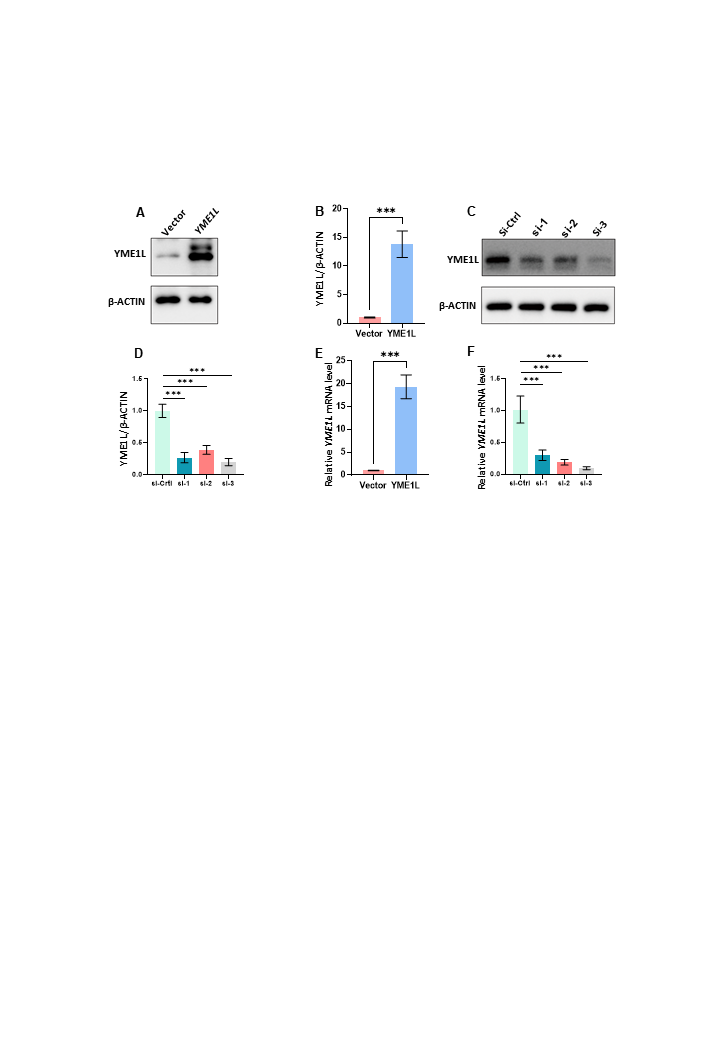
**

**Figure. S5 Effects of YME1L plasmid and siRNA transfection on the expression of YME1L in HK2 cells. (A-D) The protein level of YME1L was measured by western blotting and associated quantitative analysis in HK2 cells transfected with YME1L-plasmid, si-YME1L (n = 3). (E, F) RT-PCR analysis of YME1L mRNA expression in HK2 cells transfected with YME1L-plasmid, si-YME1L (n = 3). Data are shown as mean ± SD. ****p*<0.001.**


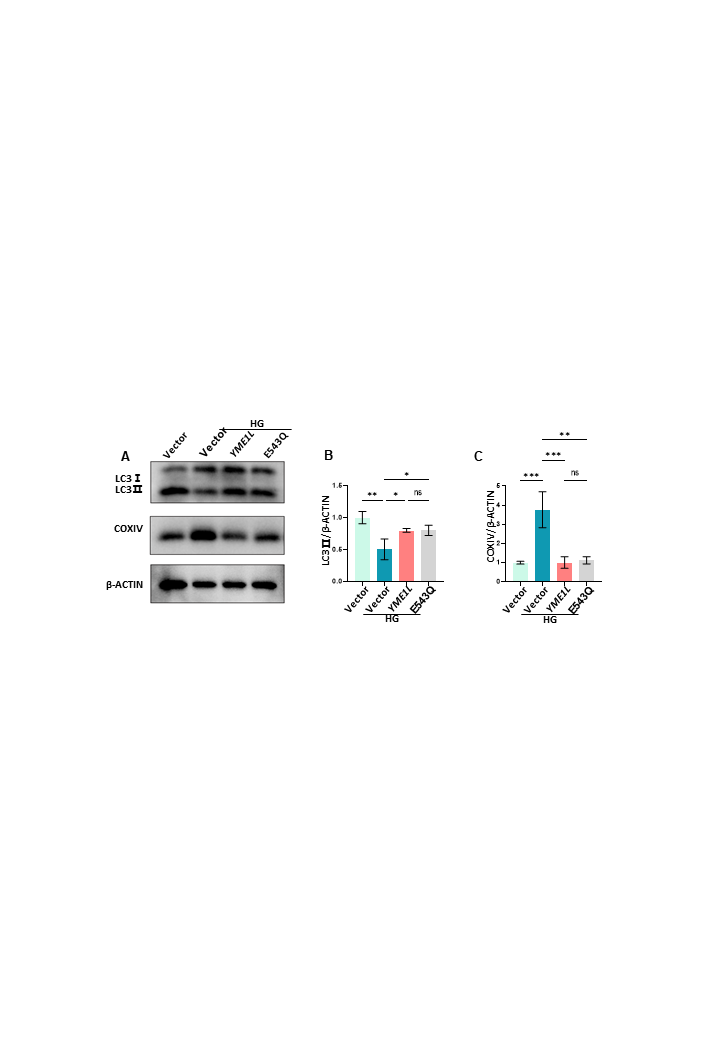


**Figure. S6 Effects of YME1L (WT)-plasmid or YME1L (E543Q) -plasmid transfection on the expression of LC3Ⅱ and COX Ⅳ in HK2 cells. (A-C) Western blotting and associated quantitative analysis of** **LC3Ⅱ and COX Ⅳ in HK2 cells transfected with YME1L (WT)-plasmid or YME1L (E543Q) -plasmid and their corresponding controls on stimulation with D-glucose for 48h (n =4). Data are shown as mean ± SD.**p*<0.05, ***p*<0.01, ****p*<0.001. ns: no statistically significant difference.**


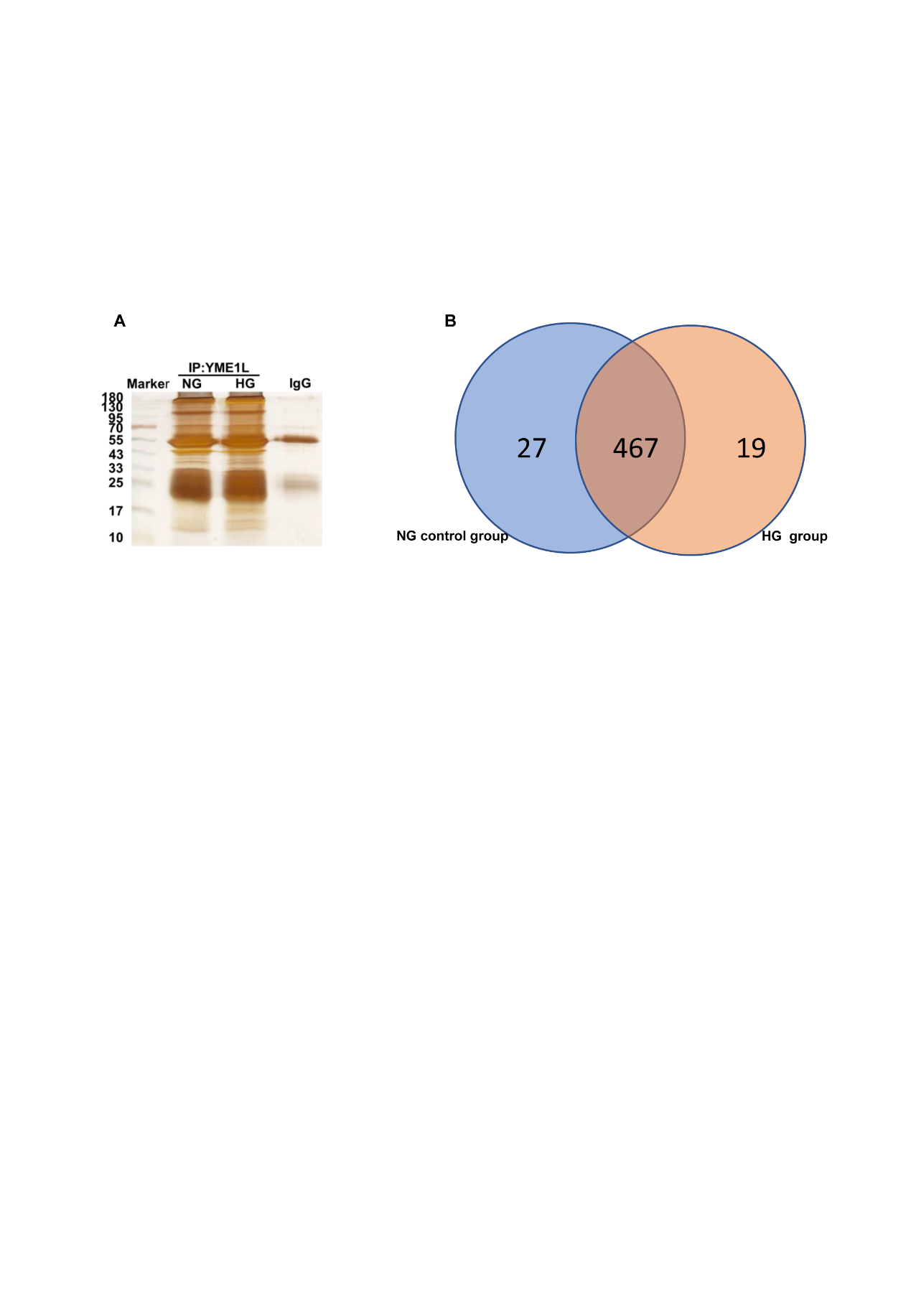


**Figure. S7 Proteomics identification, functional analysis, and validation of YME1L-interacting proteins in HK2 cells. (A) IP assay was carried out using YME1L antibody or IgG (negative control antibody). Samples were electrophoresed and silver stained. (B) Venn diagram indicating YME1L-interacting proteins in NG control and HG groups.**


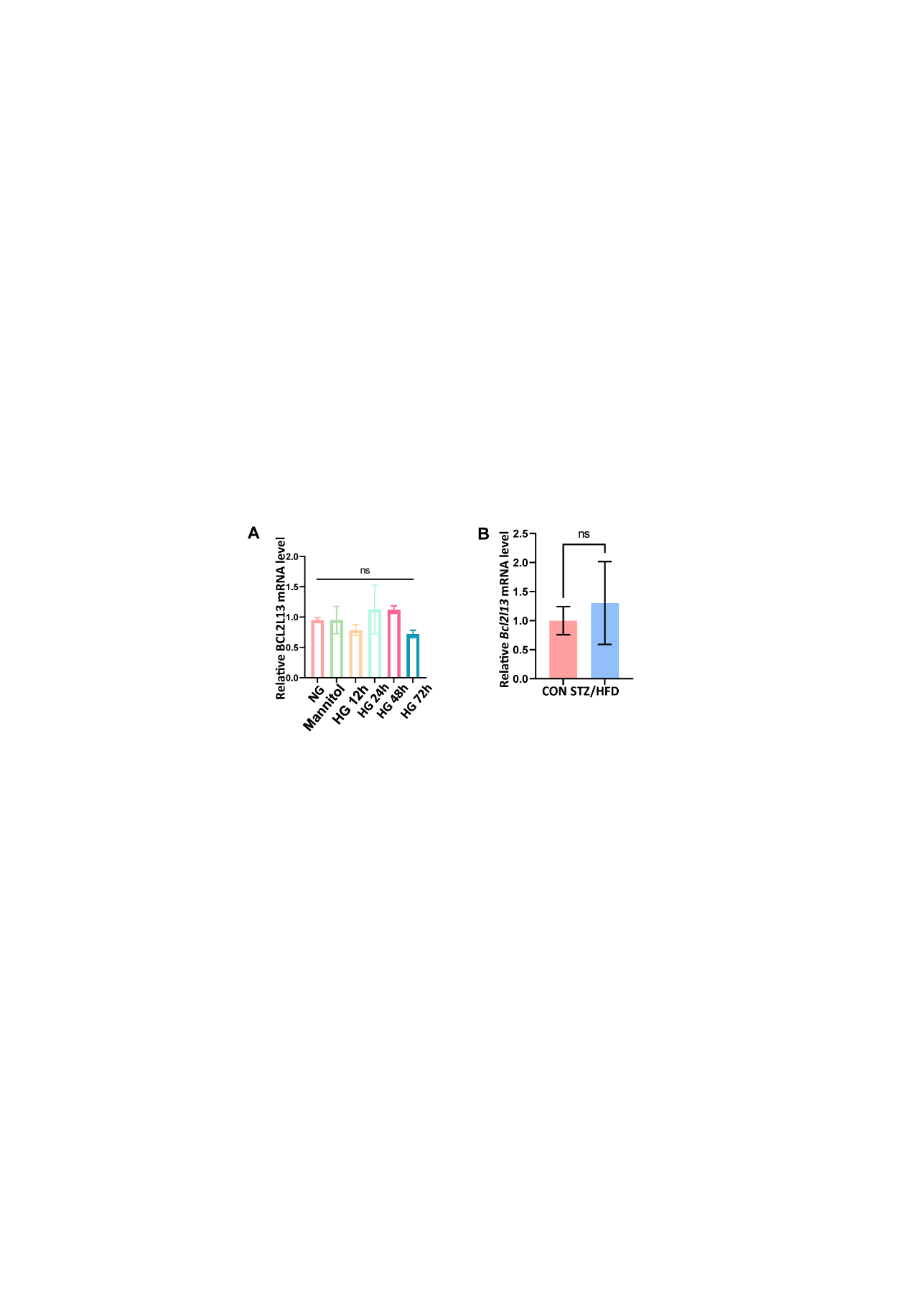


**Figure. S8 Effects of HG on transcription levels of BCL2L13 expression. (A) RT-PCR analysis of *BCL2L13* mRNA expression in HK2 cells at different time points after HG treatment (n = 3). (B) RT-PCR analysis of kidney *Bcl2l13* mRNA expression in CON and HFD/STZ mice (n = 4). Data are shown as mean ±SD. ns: no statistically significant difference.**


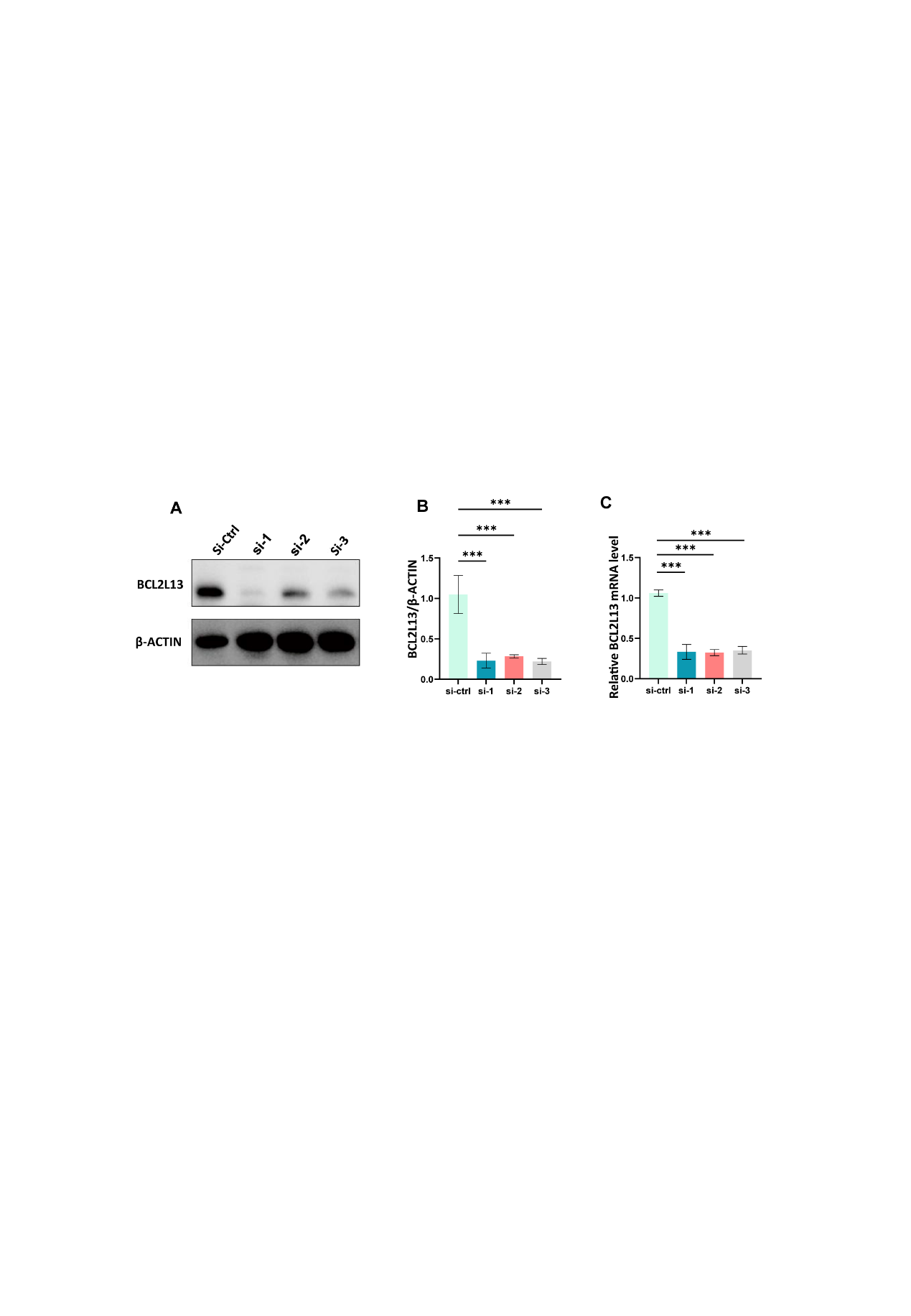


**Figure. S9 Effect of BCL2L13 siRNA transfection on the expression of BCL2L13 in HK2 cells. (A, B) The protein level of BCL2L13 was measured by western blotting and associated quantitative analysis in HK2 cells transfected with si-BCL2L13 (n = 3). (C) RT-PCR analysis of BCL2L13 mRNA expression in HK2 cells transfected with si-BCL2L13 (n = 3). Data are shown as mean ± SD. ****p*<0.001.**


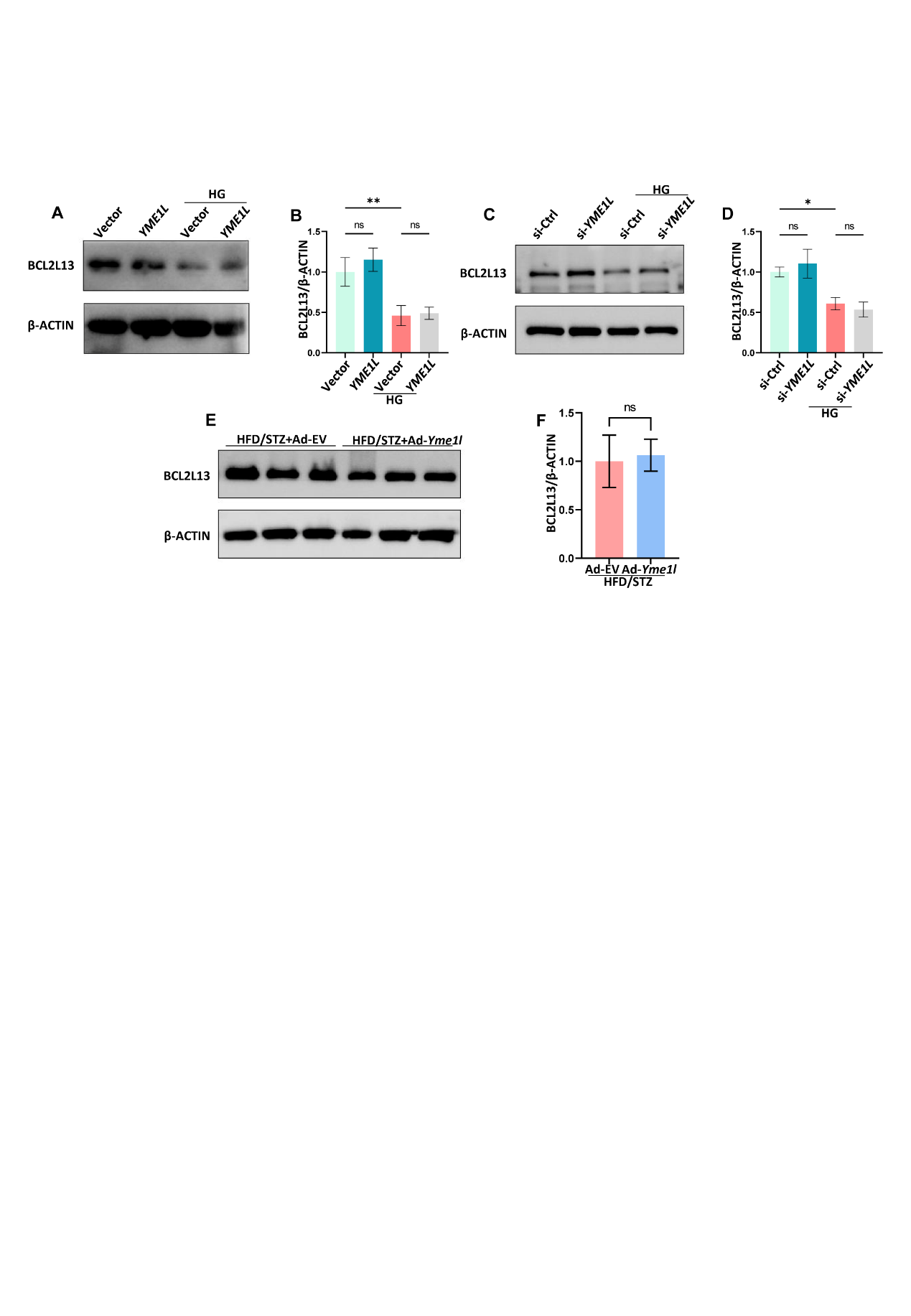


**Figure. S10 The effect of YME1L on the protein level and transcriptional level of BCL2L13. (A-D) Western blotting and associated quantitative analysis of BCL2L13 expression in HK2 cells transfected with YME1L-plasmid, si-YME1L, and their corresponding controls on stimulation with D-glucose (30 mmol/L) for 48h (n = 3). (E, F) Western blotting and associated quantitative analysis of BCL2L13 expression in HFD/STZ+Ad-EV and HFD/STZ + Ad-*Yme1l* mice (n =3). Data are shown as mean ± SD. **p*<0.05, ***p*<0.01. ns: no statistically significant difference.**
